# From macro to micro: dataset on plastic contamination along and across a sandy tide-less coast (the Curonian Spit, the Baltic Sea)

**DOI:** 10.1016/j.dib.2020.105635

**Published:** 2020-04-30

**Authors:** Elena Esiukova, Liliya Khatmullina, Olga Lobchuk, Alexey Grave, Alexander Kileso, Mirco Haseler, Andrey Zyubin, Irina Chubarenko

**Affiliations:** a Shirshov Institute of Oceanology, Russian Academy of Sciences, 36, Nakhimovski prospect, Moscow, 117997, Russia; b Immanuel Kant Baltic Federal University, 14, A. Nevskogo ul., Kaliningrad, 236016, Russia; c Leibniz Institute for Baltic Sea Research Warnemünde, Seestrasse 15, D-18119 Rostock, Germany

**Keywords:** Beach litter, Microplastics, National park, Beach zones, Curonian Spit

## Abstract

The contamination by macrolitter (>25 mm), mesolitter (5-25 mm), large microlitter (2-5 mm), large and small microplastics (L-MPs (2-5 mm) and S-MPs (0.5-2 mm), accordingly) in the surface beach sand at 6 locations along the 100-km-long marine coast of the Curonian Spit UNESCO National Park and the neighboring city beaches is quantified. In total, 55 samples obtained during 1-2 May 2018 are analyzed. Primary data is provided, along with exhaustive information on sampling dates and coordinates, sampling methods, extracting procedures, control measures, detection techniques, and μ-Raman spectroscopy verification. The number of items per m^2^ and items per kg dry weight (for MPs) is determined separately for fibres, films, and fragments. Distributions by size and plastic type are presented. Standard protocols, a modified NOAA method, and μ-Raman spectroscopy were applied to obtain the data, thus they can be used for comparative analyses.

**Specifications Table**

**Table:** 

**Subject**	Environmental Science, Ecology
**Specific subject area**	Litter, Plastic and Microplastic Contamination, Environment
**Type of data**	Table Image Chart Graph Figure
**How data were acquired**	the modified Sand Rake method [1]; a square sampling frame (18 cm × 18 cm) and stainless steel spatula [2,3]; NOAA extraction (ZnCl_2_); Stereomicroscope (Micromed MC2 Zoom Digital); Raman Centaur U (LTD “NanoScanTechnology”, Russia) spectrometer
**Data format**	Raw and Analysed
**Parameters for data collection**	Sampling of surface beach sands. Macro-, meso- and microlitter, large and small microplastics extraction according to the modified Sand Rake method and NOAA method [1], [2], [3], [4]. Contamination control. Microscopy and μ-Raman spectroscopy analyses.
**Description of data collection**	Data of the number of items per m^2^ macrolitter (>25 mm), mesolitter (5-25 mm), large microlitter (2-5 mm), large and small microplastics (L-MPs (2-5 mm) and S-MPs (0.5-2 mm) accordingly) abundances in surface beach sands on the base of 55 samples obtained at 6 locations in the expedition during 1-2 May 2018. Map of study area and sampling stations. Distribution of litter by size. Images of raw typical plastic particles and the hit ratio between the specimen spectra and reference spectra, which were identified by μ-Raman spectroscopy.
**Data source location**	The Curonian spit UNESCO National Park and the neighboring city beaches during 1-2 May 2018. 6 stations, 4 beach zones, 2 replicates.
**Data accessibility**	All data are accessible within this article.
**Related research article**	Chubarenko I., Esiukova E., Khatmullina L., Lobchuk O., Grave A., Kileso A., Haseler M. From macro to micro, from patchy to uniform: analyzing plastic contamination along and across a sandy tide-less coast. Mar. Pollut. Bull., *accepted for publication on April, 17, MPB_111198*.

**Value of the Data**

Macro-, meso- and microlitter, large and small microplastics (MPs) contamination in surface beach sands of the Curonian Spit UNESCO National Park and the neighbouring city beaches is documented.
Sampling was specially designed to grasp quasi-instant “natural” plastic contamination patterns in a large area with minor anthropogenic influence.
The idea is to develop a science-based cost-effective method for monitoring of beach plastic contamination.
Data allow for comparisons of plastic contamination along and across the National Park area. Data can be used for comparative analysis of plastic contamination in sandy beach sediments of other sandy coasts.

## 1 Data

**Fig. 1:**
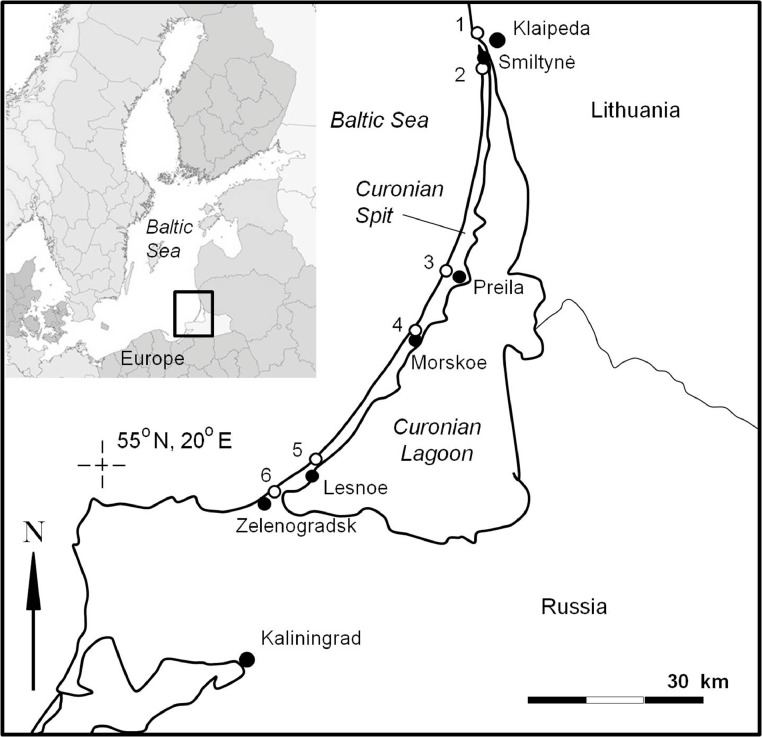
The study area in the southeastern Baltic Sea. Sampling locations are indicated by white circles, the closest villages (all located at the lagoon site) – by black circles.

**Fig. 2:**
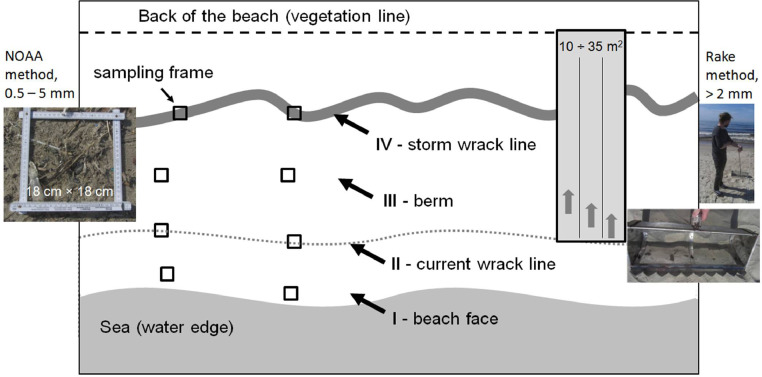
The sampling scheme, repeated at every location: raking for litter objects > 2 mm and sampling nearby for MPs. The raking area at different locations varied between 10 and 35 m^2^ (see Table 1, Appendix 1). The zones of the beach and the scheme of sampling for MPs are shown: (I) the beach face, (II) the first (current) wrack line, (III) the middle part of the winter berm, and (IV) the strongest winter-storm wrack line; two replicates ca. 5 m apart were taken in every beach zone.

**Fig. 3:**
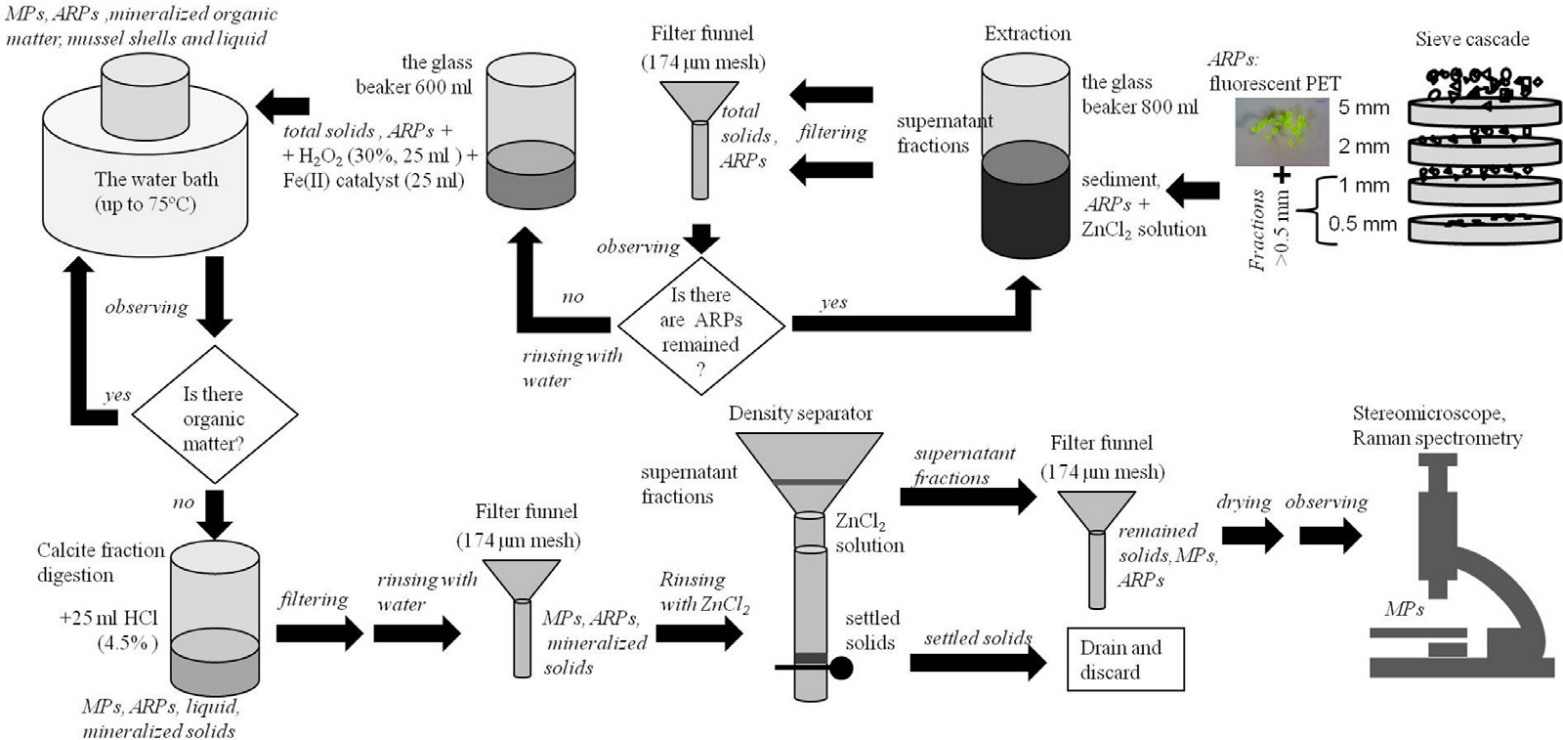
Analysis procedures: the modified NOAA method.

**Fig. 4:**
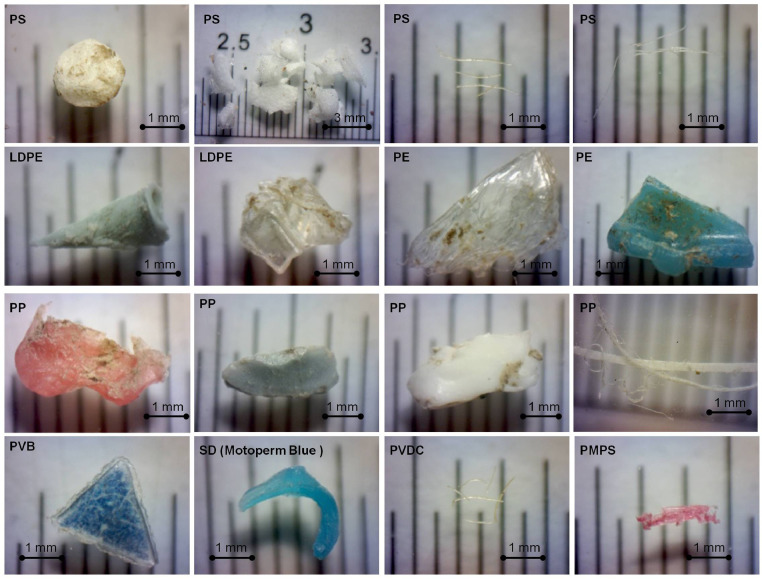
Examples of MPs particles found in this study.

The dataset contains information about macrolitter (>25 mm), mesolitter (5-25 mm), large microlitter (2-5 mm), large and small microplastics (L-MPs (2-5 mm) and S-MPs (0.5-2 mm) accordingly) concentration in 55 sandy beach sediments samples collected at 6 locations along the 100-km-long marine coast of the Curonian Spit UNESCO National Park (located in-between the cities of Klaipeda (Lithuania) and Zelenogradsk (Russia)) and the neighboring cities during 1-2 May 2018. The study site (Fig. 1), geographic reference, and general characteristics of sampling locations and sample characteristics are presented in (Table 1). The sampling scheme at every location is presented in (Fig. 2). The data of Sand Rake method [1] for macro-, meso- and large microlitter monitoring are presented in all commonly used units: number of items in a sample, number of items per square meter (items per m^2^), and number of items per m of the coast length (Table 2). The data of the square sampling frame method for MPs monitoring for two size classes (S-MPs (0.5-2 mm) and L-MPs (2-5 mm)) from 4 beach zones are presented in the number of items in a sample, the number of items per square meter (items per m^2^), and the number of items per kg dry weight (items per kg DW) (Table 3). The laboratory analysis procedures are presented in (Fig. 3). The photos of twelve selected MPs specimens extracted from the sediments are presented in (Fig. 4). The polymer types identified with Raman spectroscopy are presented in (Table 4), and the types of polymers in three groups (shapes) of MPs (in percent) are presented in (Table 5).

**Table 1:** Dates, sampling sites locations and general characteristics.

№ sampling stations	Country	Date	Location region	Latitude	Longitude	Width of the beach, m	Sand Rake Method			Square sampling frame method	
							Sampling area, m^2^	Number of stripes (0.5 m)	Number of sections in stripe (5 m each)	Number of samples	Number of beach zones
**1**	Lithuania	May 1, 2018	Klaipeda	55.73098333	21.08517667	20	10	1	4	8	4
**2**	Lithuania	May 2, 2018	Smiltynė	55.67671	21.103	55	27.5	1	11	9	4
**3**	Lithuania	May 2, 2018	Preila	55.37723333	21.03051667	35	30	2	14	8	4
**4**	Russia	May 2, 2018	Morskoe	55.23906	20.90783333	65	32.5	1	13	8	4
**5**	Russia	May 2, 2018	Lesnoe	55.030305	20.63372833	35	35	2	14	9	4
**6**	Russia	May 2, 2018	Zelenogradsk	54.967667	20.495685	18	-	-	-	8	4

**Table 2:** Data of the Sand Rake method for macrolitter (>25 mm), mesolitter (5-25 mm), large microlitter (2-5 mm) monitoring: total number of items found, items per square meter (items per m^2^), and items per 1 m of the coast length (items per m).

Location	Size	Cigarettes	Plastic	Paper	Metal	Glass / Ceramics	Rubber	Paraffin	Wood	Total	Bulk concentration, items per m^2^ / items per m
Klaipeda	microlitter		149	1				1		151	
	mesolitter	6	54	3	1			2		66	
	macrolitter	2	29						1	32	
	**Total**	**8**	**232**	**4**	**1**	**0**		**3**	**1**	**249**	24.90 / 498

Smiltynė	microlitter		23				1	6		30	
	mesolitter		6	1				1	1	9	
	macrolitter		9	1					1	11	
	**Total**	**0**	**38**	**2**	**0**	**0**	**1**	**7**	**2**	**50**	1.82 / 100

Preila	microlitter		8					5		13	
	mesolitter	3	9	2						14	
	macrolitter		8							8	
	**Total**	**3**	**25**	**2**	**0**	**0**	**0**	**5**	**0**	**35**	1.17 / 35

Morskoe	microlitter		22					7		29	
	mesolitter		21			2		8		31	
	macrolitter		15			1				16	
	**Total**	**0**	**58**	**0**	**0**	**3**	**0**	**15**	**0**	**76**	2.34 / 152

Lesnoe	microlitter		3					2	0	5	
	mesolitter	1	3	1		4		3	0	12	
	macrolitter		4		1				0	5	
	**Total**	**1**	**10**	**1**	**1**	**4**		**5**	**0**	**22**	0.63 / 22

**Table 3:** Data of the square (18 cm × 18 cm) sampling frame method for total MPs, and separately for two size classes (S-MPs (0.5-2 mm) and L-MPs (2-5 mm)) from 4 beach zones (in 2 replicates): (i) the number of items in a sample (*items*), (ii) the number of items per square meter (*items per m^2^*), and (iii) the number of items per kg dry weight (*items per kg DW*).

Location	Beach zone, sample number	S-MPs (0.5-2 mm), *items*	S-MPs (0.5-2 mm), *items per m^2^*	S-MPs (0.5-2 mm), *items per kg DW*	L-MPs (2-5 mm), *items*	L-MPs (2-5 mm), *items per m^2^*	L-MPs (2-5 mm), *items per kg DW*	MP total (0.5-5 mm), *items*	MP total (0.5-5 mm), *items per m^2^*	MP total (0.5-5 mm), *items per kg DW*
Klaipeda	storm wrack line 4/1	60	1846	132	3	92	7	63	1938	139
	storm wrack line 4/2	92	2831	244	0	0	0	92	2831	244
	berm 3/1	17	523	29	0	0	0	17	523	29
	berm 3/2	44	1354	85	0	0	0	44	1354	85
	current wrack line 2/1	25	769	34	0	0	0	25	769	34
	current wrack line 2/2	105	3231	123	0	0	0	105	3231	123
	beach face 1/1	19	585	28	0	0	0	19	585	28
	beach face 1/2	117	3600	96	0	0	0	117	3600	96
Smiltynė	storm wrack line 4/1	172	5292	292	3	92	5	175	5385	297
	storm wrack line 4/2	180	5538	292	5	154	8	185	5692	300
	berm 3/1	391	12031	215	0	0	0	391	12031	215
	berm 3/2	126	3877	72	0	0	0	126	3877	72
	current wrack line 2/1	123	3785	176	1	31	1	124	3815	177
	current wrack line 2/2	14	431	13	1	31	1	15	462	14
	beach face 1/1	27	831	17	0	0	0	27	831	17
	beach face 1/2	121	3723	80	0	0	0	121	3723	80
Preila	storm wrack line 4/1	30	923	35	2	62	2	32	985	38
	storm wrack line 4/2	117	3600	118	0	0	0	117	3600	118
	storm wrack line 4/3	897	27600	1338	3	92	4	900	27692	1343
	berm 3/1	204	6277	131	0	0	0	204	6277	131
	berm 3/2	507	15600	779	0	0	0	507	15600	779
	current wrack line 2/1	31	954	40	0	0	0	31	954	40
	current wrack line 2/2	25	769	29	0	0	0	25	769	29
	beach face 1/1	115	3538	84	0	0	0	115	3538	84
	beach face 1/2	137	4215	111	0	0	0	137	4215	111
Morskoe	storm wrack line 4/1	154	4738	114	1	31	1	155	4769	115
	storm wrack line 4/2	11	338	9	2	62	2	13	400	11
	berm 3/1	24	738	19	0	0	0	24	738	19
	berm 3/2	33	1015	17	0	0	0	33	1015	17
	current wrack line 2/1	13	400	14	0	0	0	13	400	14
	current wrack line 2/2	8	246	7	2	62	2	10	308	9
	beach face 1/1	7	215	5	0	0	0	7	215	5
	beach face 1/2	15	462	12	0	0	0	15	462	12
Lesnoe	storm wrack line 4/1	23	708	19	0	0	0	23	708	19
	storm wrack line 4/2	38	1169	32	0	0	0	38	1169	32
	storm wrack line 4/3	138	4246	120	0	0	0	138	4246	120
	berm 3/1	65	2000	44	0	0	0	65	2000	44
	berm 3/2	116	3569	71	0	0	0	116	3569	71
	current wrack line 2/1	34	1046	32	0	0	0	34	1046	32
	current wrack line 2/2	8	246	9	0	0	0	8	246	9
	beach face 1/1	66	2031	42	0	0	0	66	2031	42
	beach face 1/2	111	3415	64	0	0	0	111	3415	64
Zelenogradsk	storm wrack line 4/1	218	6708	240	0	0	0	218	6708	240
	storm wrack line 4/2	102	3138	88	1	31	1	103	3169	89
	berm 3/1	12	369	8	0	0	0	12	369	8
	berm 3/2	13	400	8	0	0	0	13	400	8
	current wrack line 2/1	51	1569	47	1	31	1	52	1600	48
	current wrack line 2/2	6	185	5	0	0	0	6	185	5
	beach face 1/1	8	246	8	0	0	0	8	246	8
	beach face 1/2	132	4062	114	0	0	0	132	4062	114

**Table 4:** Polymer type and types of synthetic dyes identified using μ-Raman spectroscopy.

	Polymer type	Acronym	%	Types of Synthetic Dyes (SD):
1	Polyethylene	PE	30.0	Hostasol-Green G-K
2	Polypropylene	PP	17.1	Motoperm Blue
3	Polystyrene	PS	11.4	Pigment red
4	Strong background fluorescence	fluorescence	10.0	Van Duke Brown
5	Low density polyethylene	LDPE	8.6	Amido Black 10B
6	Synthetic dyes	SD	4.3	Cobalt phthalocyanine
7	Cellulose/Cellulose acetate	CE/CA	2.9	Astra Blue Base
8	Polyethylene terephthalate/Polyester	PET/PES	2.9	
9	Plastic wax	Plastic wax	2.9	
10	Polyvinyl chloride acetate	PVCA	2.9	
11	Nylon 6	Nylon	1.4	
12	Polymethylphenylsiloxane	PMPS	1.4	
13	Polyvinyl acetate	PVA	1.4	
14	Polyvinyl Butiral	PVB	1.4	
15	Polyvinylidene chloride	PVDC	1.4

**Table 5:** Types of polymers in three groups (shapes) of microplastics (in percent).

	Percentage from items in each individual group (shape), %				Percentage of total number of identified particles, %			
	Fragments	Films	Fibres		Fragments	Films	Fibres	SUM, %
PE	31.4	54.5	16.7	PE	15.7	8.6	5.7	30.0
PP	22.9	0.0	16.7	PP	11.4	0.0	5.7	17.1
PS	8.6	0.0	20.8	PS	4.3	0.0	7.1	11.4
fluorescence	2.9	18.2	16.7	fluorescence	1.4	2.9	5.7	10.0
LDPE	17.1	0.0	0.0	LDPE	8.6	0.0	0.0	8.6
SD	5.7	9.1	0.0	SD	2.9	1.4	0.0	4.3
CE/CA	2.9	0.0	4.2	CE/CA	1.4	0.0	1.4	2.9
PET/PES	0.0	0.0	8.3	PET/PES	0.0	0.0	2.9	2.9
Plastic wax	2.9	9.1	0.0	Plastic wax	1.4	1.4	0.0	2.9
PVCA	0.0	0.0	8.3	PVCA	0.0	0.0	2.9	2.9
Nylon	2.9	0.0	0.0	Nylon	1.4	0.0	0.0	1.4
PMPS	0.0	9.1	0.0	PMPS	0.0	1.4	0.0	1.4
PVA	0.0	0.0	4.2	PVA	0.0	0.0	1.4	1.4
PVB	2.9	0.0	0.0	PVB	1.4	0.0	0.0	1.4
PVDC	0.0	0.0	4.2	PVDC	0.0	0.0	1.4	1.4
SUM, %	100	100	100	SUM, %	50.0	15.7	34.3	100.0

The dataset containing a detailed information about macro-, meso- and microlitter and large and small MPs contamination for each station in MS Excel format is provided in Supplementary Material (Appendix 1). The data on identification of S-MPs (0.5-2 mm) by μ-Raman spectroscopy are presented in Appendix 2. The polymer types, types of synthetic dyes, images of MPs, the hit ratio between the specimen spectra and reference spectra, which were identified by μ-Raman spectroscopy, are presented in Appendix 3.

## 2 Experimental Design, Materials, and Methods

### 2.1 Sediment sampling

The samples were collected at 6 locations along the 100-km-long marine coast of the Curonian Spit UNESCO National Park (located in-between the cities of Klaipeda (Lithuania) and Zelenogradsk (Russia)) and the neighboring city beaches in the southeastern Baltic Sea during 1-2 May 2018 (Fig. 1). The sand samples for analysis of L-MPs (2-5 mm) and S-MPs (0.5-2 mm) content were collected at 6 locations along the coast (4 beach zones, in 2 replicates each), while the abundance of macrolitter (>25 mm), mesolitter (5-25 mm), and microlitter (2-5 mm) was quantified only at 5 of them, due to weather conditions. Two sampling methods were simultaneously applied: the Sand Rake method for litter larger than 2 mm [1], and the sampling frame method for MPs (see [2,3]) for MPs (0.5-5 mm). Throughout the text, we keep the exact meaning of the terms for anthropogenic debris items: macro-, meso-, and microlitter include all anthropogenic items (glass, paper, ceramics, plastic, etc), while macro-, meso-, and microplastic is solely plastic.

Anthropogenic (both plastic and non-plastic) litter in the surface 3–5 сm of the beach sediments was quantified directly on-site by the modified Sand Rake method [1]. Following this method, debris was collected from the entire width of the beach (from 25 to 65 m) between the waterline (current wrack line in Fig. 2) and the vegetation line / cliff using a metallic rake with the mesh size of 2 mm (see photo on the right-hand side of Fig. 2). The exact location of the sampling sections at the coastline was chosen randomly since wide and flattened beaches under investigation did not show evident topographic peculiarities or large litter patches. Raking was impossible at St. 6 (Zelenogradsk): sands became wet due to heavy rain. The total raked area amounts to 135 m^2^. All the collected litter was further divided by fractions and analyzed in the laboratory.

The sand samples for analysis on MPs (0.5-5 mm) content were collected from four zones across the beach, with two replicates (about 5 m apart) in each zone (Fig. 2): the beach face, the current wrack line, the middle of the winter berm, and the wrack line left after the past storm. The sand sediments were collected from the upper 2-cm layer using a wooden square sampling frame (18 cm × 18 cm) and a clean stainless steel spatula. In total, 50 samples were collected by this method, making an integral sampled area of 1.625 m^2^. All the sand samples were packed into new polyethylene bags with a string lock, and transported into the laboratory for further analysis.

### 2.2 Methods

#### 2.2.1 Sample Preparation

Microplastics were extracted from the beach sand samples using the method employed in [4] with modifications [3,5]. Initial steps included drying, weighing and sieving the samples through the cascade of four sieves (mesh sizes of 5, 2, 1, and 0.5 mm). Visually detected MPs (as well as organic debris, amber, glass, paraffin, etc.) were removed directly from the sieves, while the residue remaining between the sieves 2 and 0.5 mm was treated using the modified NOAA method for the extraction of MPs from a sediment sample (see [2,3,5,6]), developed on the base of the NOAA recommendations [4]. It includes (I) density separation in the solution of ZnCl_2_ (density 1.6 g mL^−1^), filtering (174 μm), wet peroxide oxidation (H_2_O_2_ (30%) at 75 ^о^С), calcite fraction removal by HCl solution; (II) once again - filtering (174 μm), density separation (1.6 g mL^−1^), filtering (174 μm), (III) examination under a stereomicroscope (Micromed MС2 Zoom Digital) with the magnification from 10 × to 40 × directly on the surface of the filter according to [7], and (IV) MPs identification with a Raman spectrometer (Fig. 3). The extracted microparticles were classified into three generic groups: fragments, films, and fibers according to [8].

#### 2.2.2 Analytical techniques

Larger particles were picked up, and “plastics” were identified visually, with the aid of a UV-lamp, mechanical stretching, and testing by hot needle, according to the recommendations for the microscopic determination [7]. The extracted small microparticles were optically analyzed and photographed using a stereomicroscope (Micromed MC2 Zoom Digital) with magnification from × 10 to × 40, and a UV-lamp was used when required (similar to the process described in [3]). The single operator performed all the detection and analysis procedures to exclude inter-operator variability. Raman spectroscopy was used to verify the result and attain the composition of plastic-like particles [9]. A Raman Centaur U (LTD “NanoScanTechnology”, Russia) spectrometer was used to obtain plastic spectra [10,11].

#### 2.2.3 Contamination and quality controls

All instruments used during the extraction process were washed with distilled water and dried before the analysis. Along with usual caution to prevent the external contamination of the samples (cotton clothes, glass/metal containers, metal laboratory equipment, glass tableware), quality control measures were applied whenever possible: control white paper sheets were disposed in working space during all the time of sample handling to estimate possible contamination from laboratory air. Fifty blank samples were run to assess the level of background contamination. The numbers of fibers in controls was not statistically significant compared with MPs concentration found in samples.

Artificial reference particles (ARPs) were added to each sample prior to the extraction procedure as an additional measure to control the extraction efficiency. A detailed description of this effective method of extraction control is provided [3,6,10,11].

#### 2.2.4 Verification by μ-Raman spectroscopy

In order to maximize the verification efficiency, the procedure of preliminary analysis and particle sorting was applied. The items for verification were selected not randomly, but as representatives for larger groups of particles, similar by their visual appearance (shapes, colours), mechanical quality (rigid, soft, elastic, foamed, etc.), and behaviour during the hot-needle test. In total, out of 5102 items (0.5-2 mm) found in sand samples, 85 items (about 2%) were selected for verification by Raman spectrometry. From them, for example, only 2 items of polystyrene foam fragments were selected out of 714 similar items, 22 coloured fibers out of 1048 similar ones, 6 out of 39 coloured films, etc. (Appendix 2).

The analysis procedure followed [10]. The polymer type and types of synthetic dyes identified using μ-Raman spectroscopy are presented in Table 4 and Table 5. In other cases, the core polymer type of some specimens was impossible to identify because of the strong signal induced by strong background fluorescence, by synthetic dyes (SD) or chemical compounds remaining on the surface of a particle. Still, the fact of the presence of SD was considered as confirmation of the synthetic origin of a particle. So, all such specimens were accounted for as MPs (see photos in Fig. 4).

## Declaration of Competing Interest

The authors declare that they have no known competing financial interests or personal relationships that could have appeared to influence the work reported in this paper.

## Appendix Supplementary materials

Image, application 1
