# Supplementary material for: From macro to micro: dataset on plastic contamination along and across a sandy tide-less coast (the Curonian Spit, the Baltic Sea)
Source: Data Brief. 2020 Apr 30;30:105635. doi: 10.1016/j.dib.2020.105635 (PMC7214824; doi:10.1016/j.dib.2020.105635)
Supplement: Supplementary file 1 [file mmc1.zip › Appendix 3_Polymer types.pdf]

Appendix 3 - Polymer types, μ-Raman spectroscopy

Polymer types	Acronym	The hit ratio between the specimen spectra and reference spectra	Established types of Synthetic Dyes (SD)	Images of MPs
**Polystyrene**	**PS**	**94**		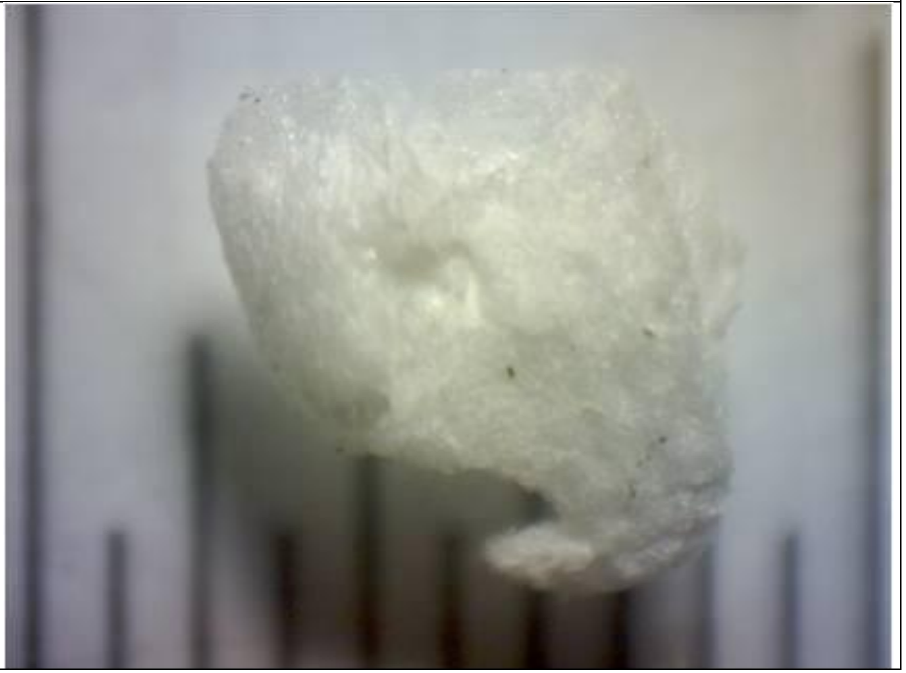

**Polystyrene**	**PS**	**98**		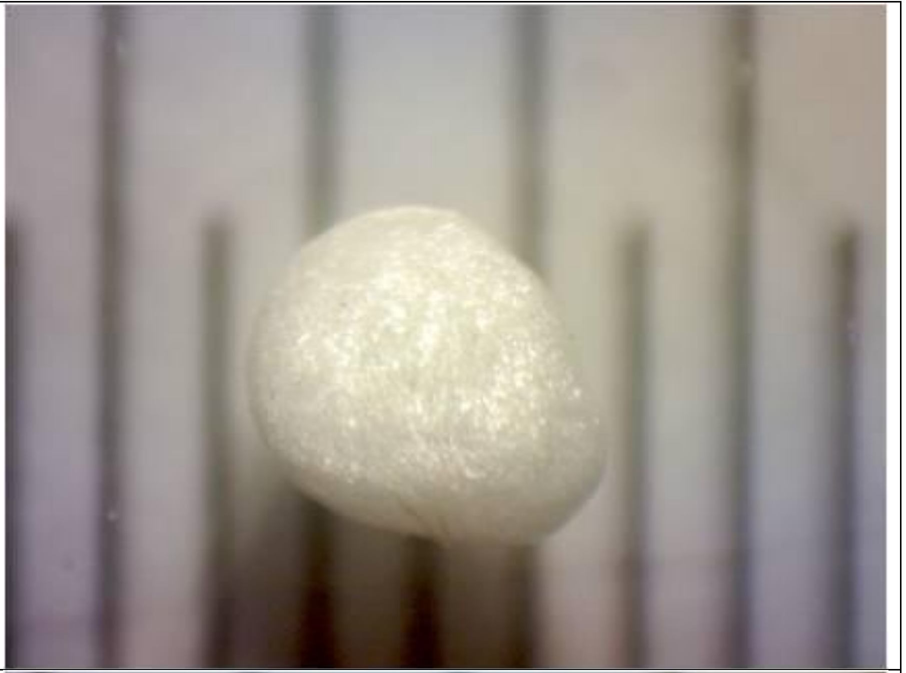
**Polyethylene**	**PE**	**91**	**Van Duke Brown**	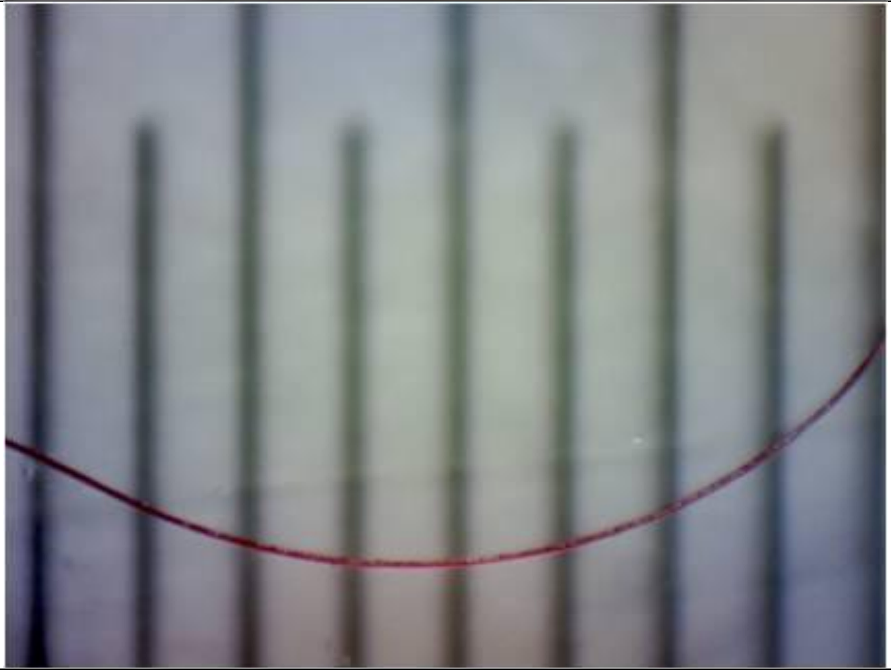

**Polystyrene**	**PS**	**76**		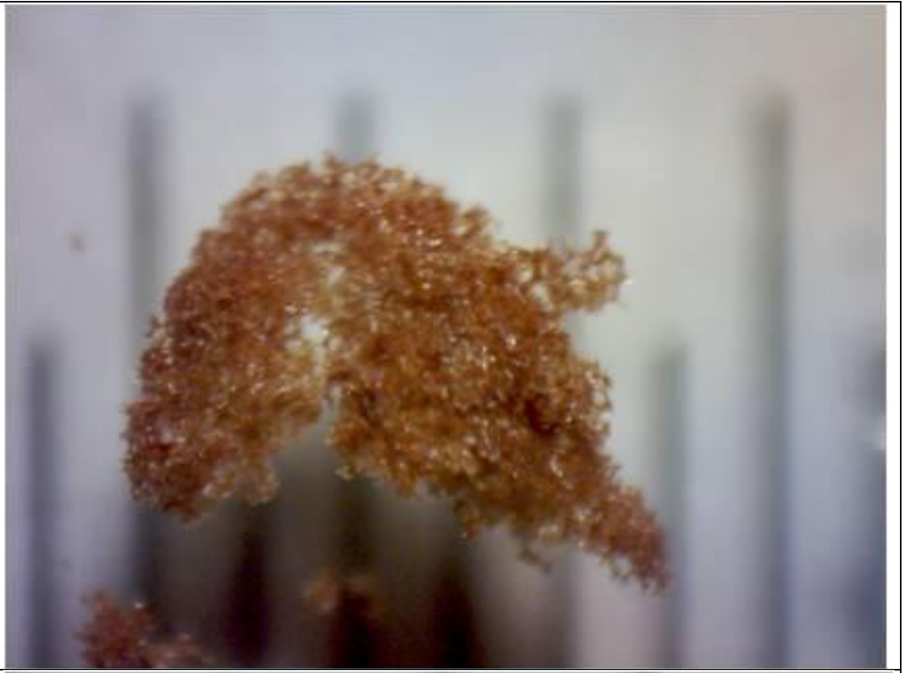
**Polypropylene**	**PP**	**85.9**		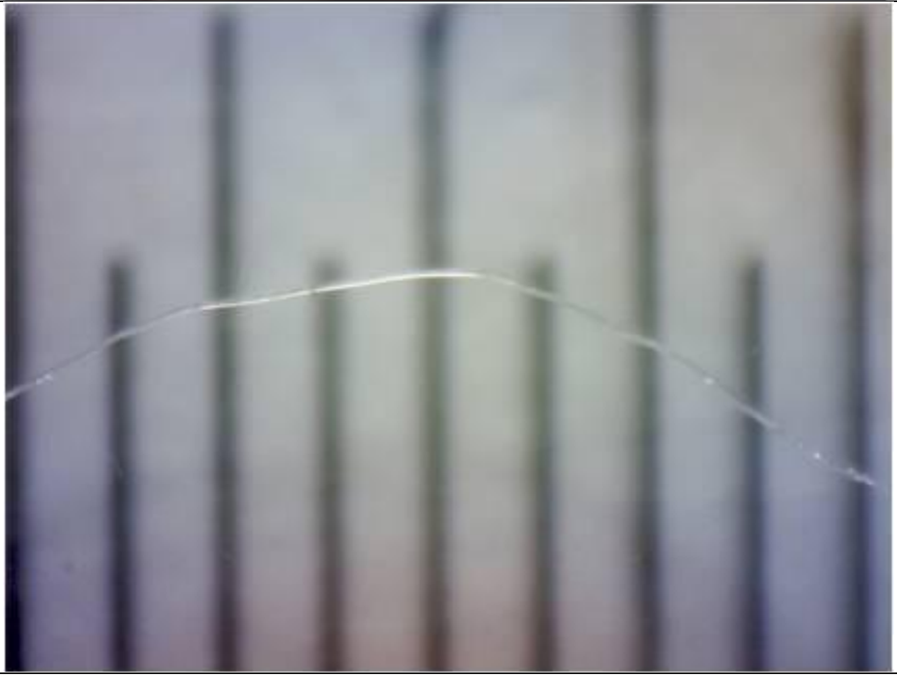

**Polyvinyl acetate**	**PVA**	**69.6**		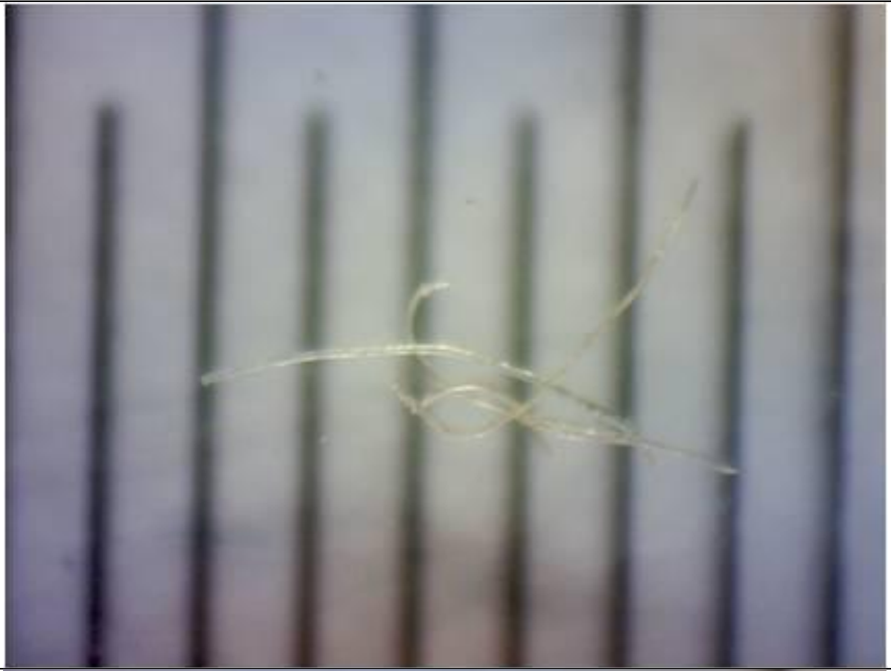
**Polystyrene**	**PS**	**63**		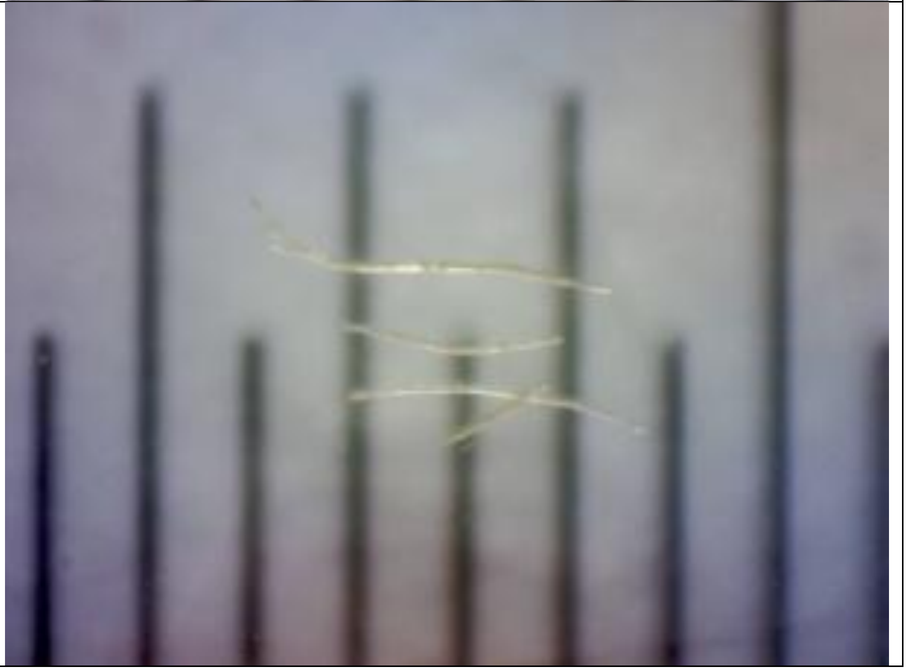

**Polyethylene terephthalate/Polyester**	**PET/PES**	**86.4**		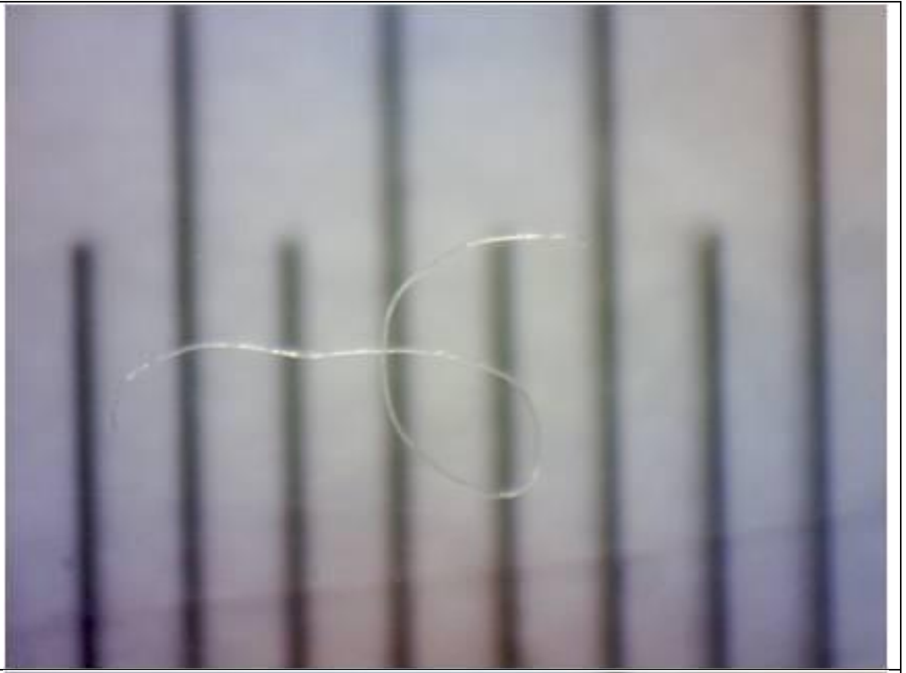
**Polyethylene terephthalate/Polyester**	**PET/PES**	**87.2**		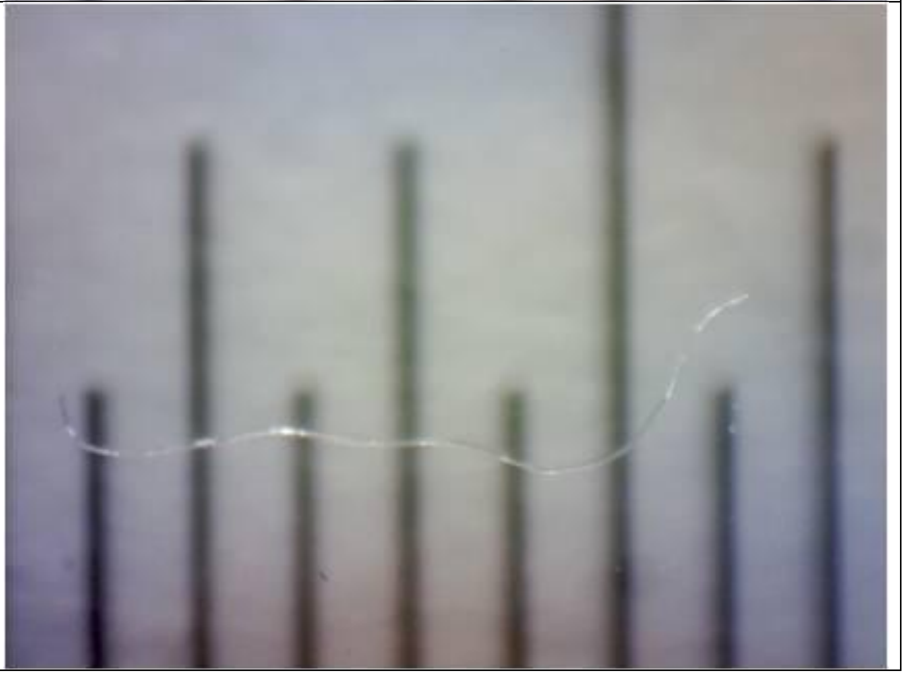

**Polyvinyl chloride acetate**	**PVCA**	**51.3**		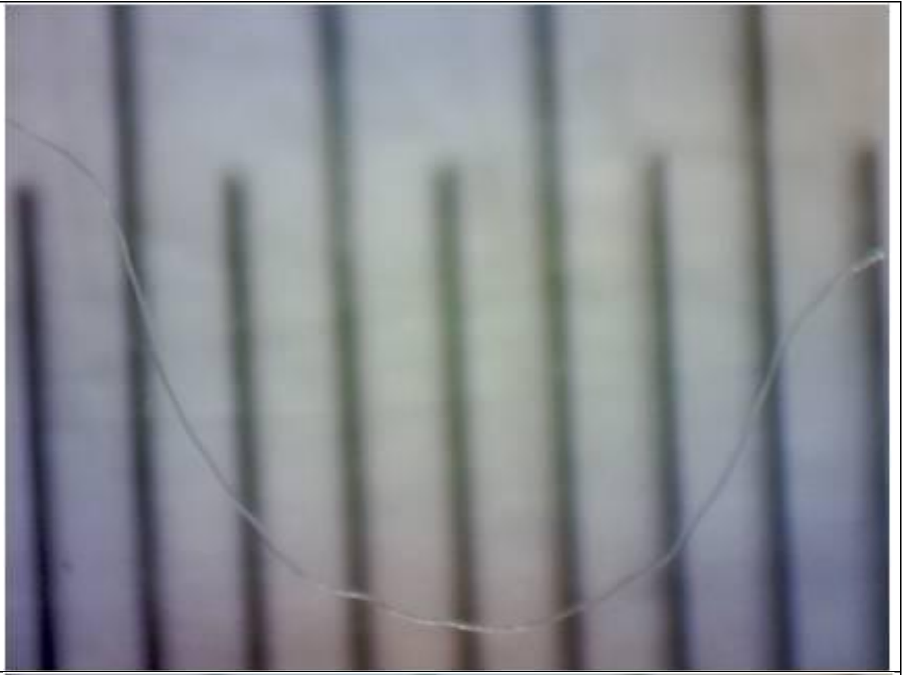
**Strong background fluorescence**	**fluorescence**			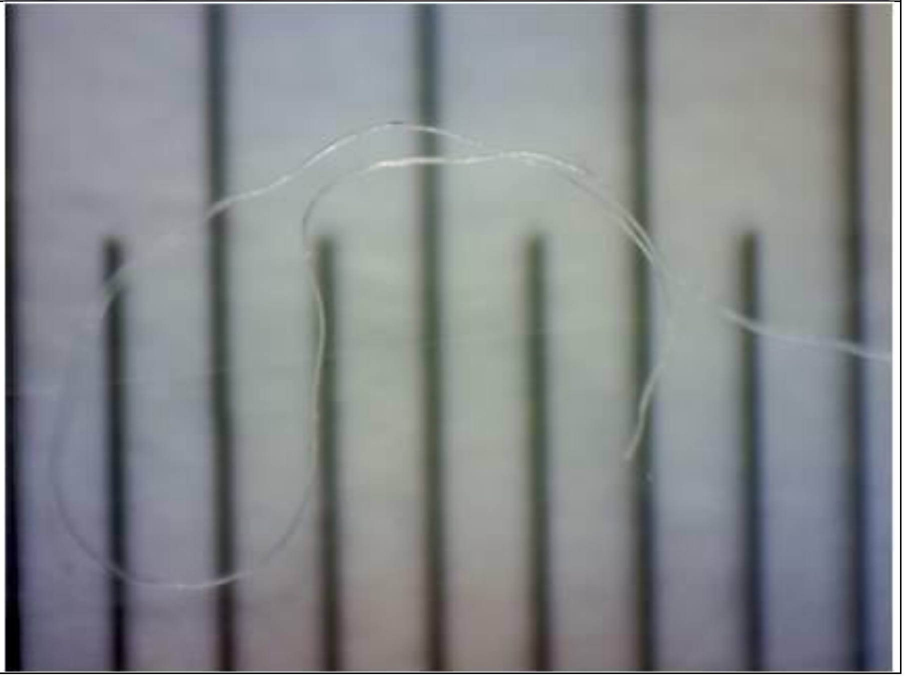

**Cellulose**	**CE**	**65.5**		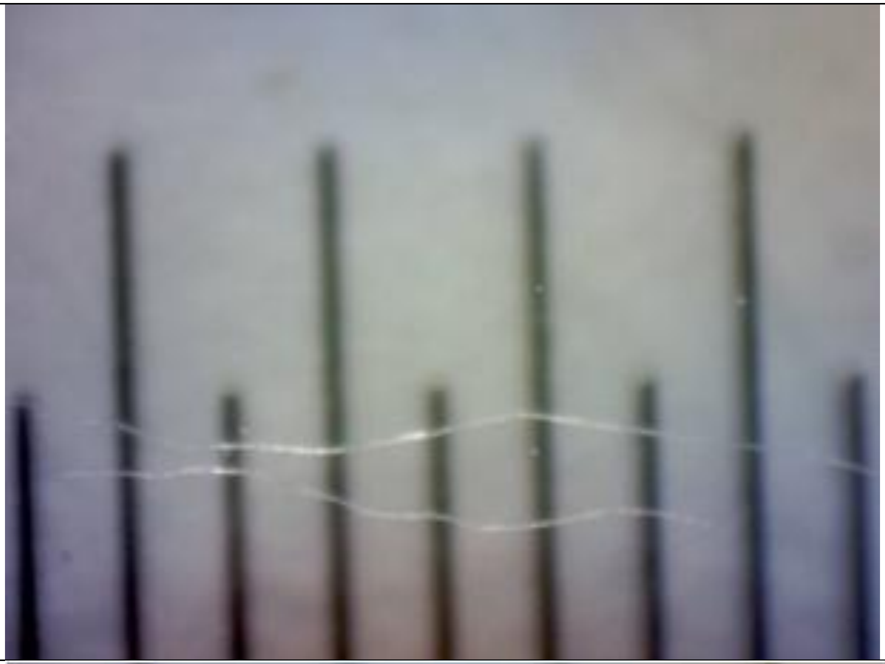
**Polyethylene**	**PE**	**75.1**	**Pigment red**	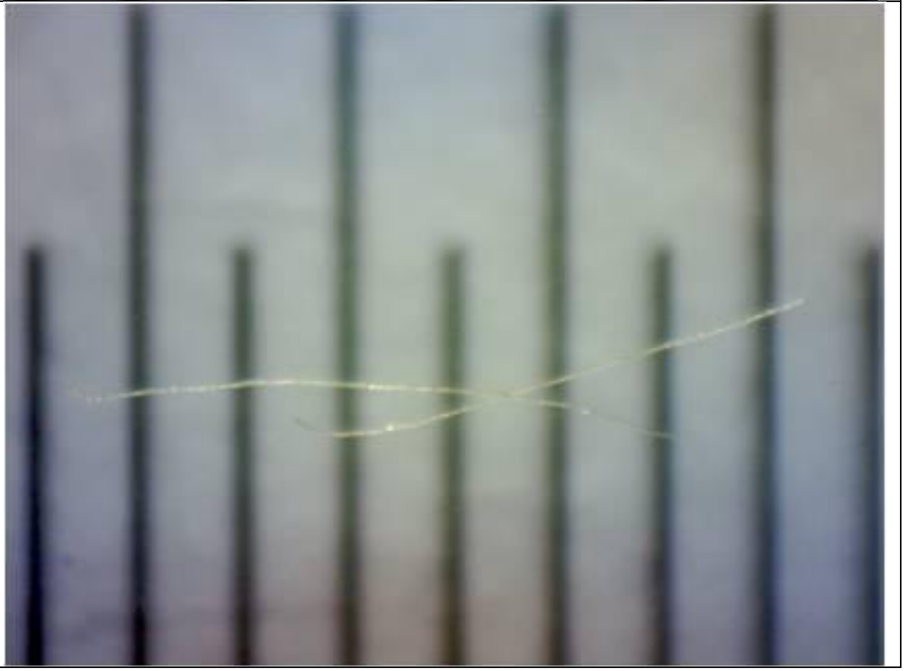

**Polystyrene**	**PS**	**68**		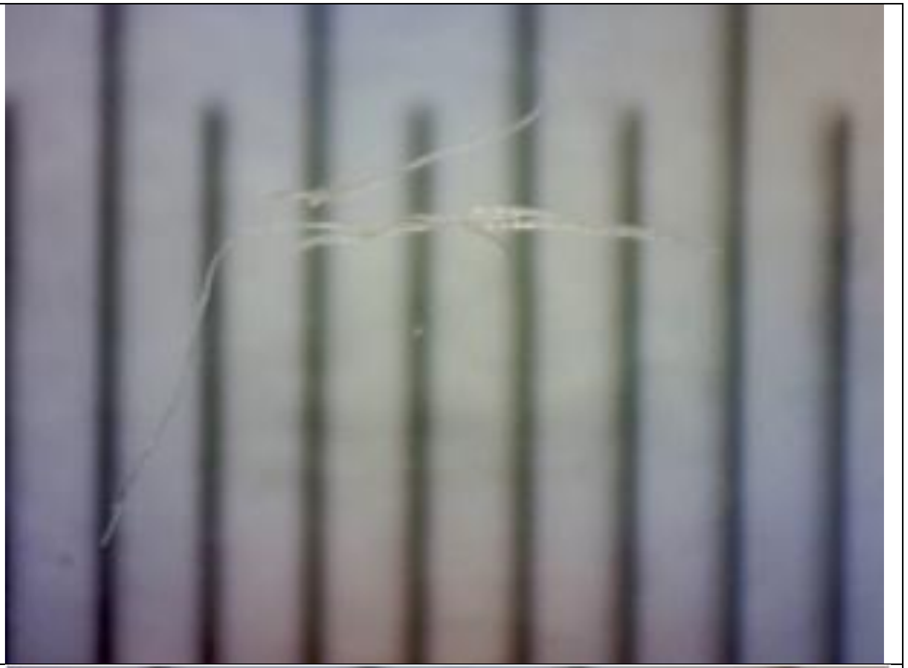
**Polypropylene**	**PP**	**85.1**	**Amido Black 10B**	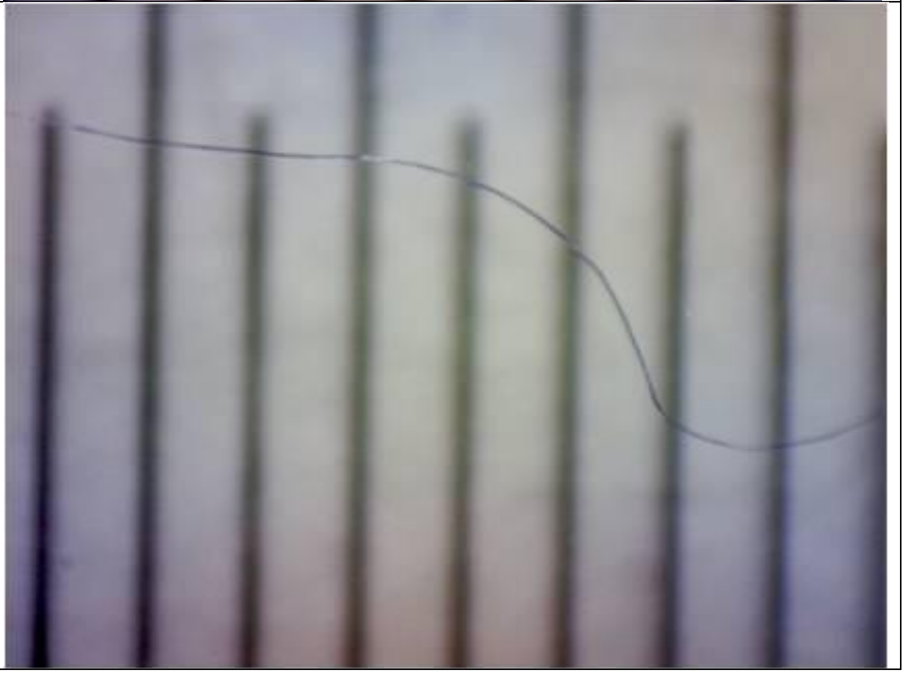

**Polystyrene**	**PS**	**78**		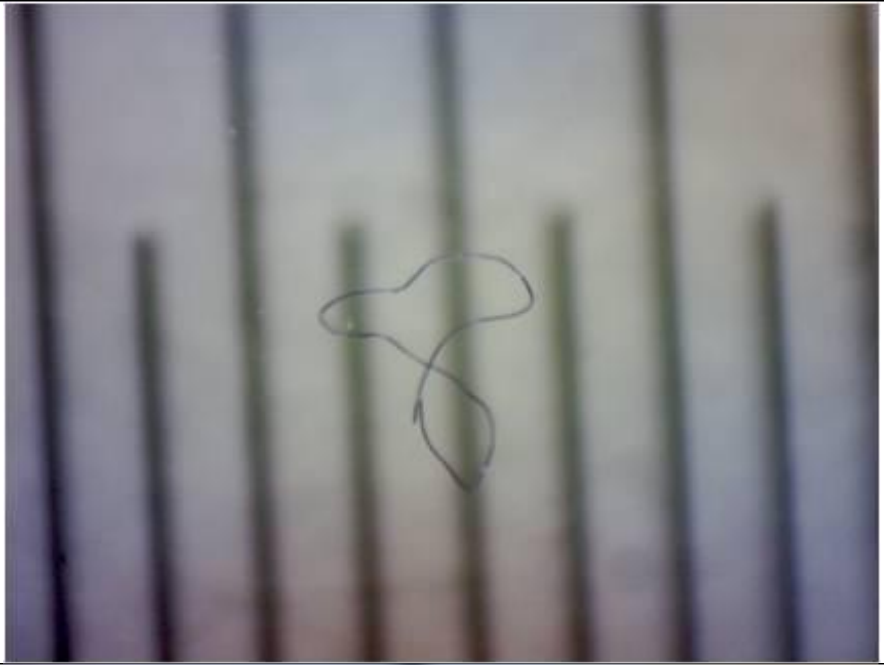
**Polystyrene**	**PS**	**72**	**Amido Black 10B**	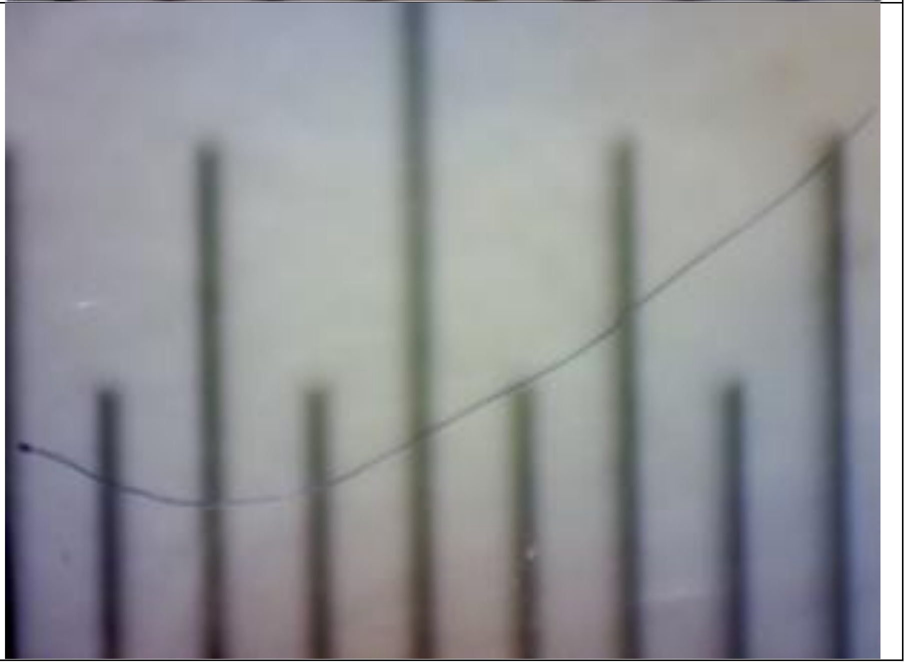

**Polyvinyl chloride acetate**	**PVCA**	**42**		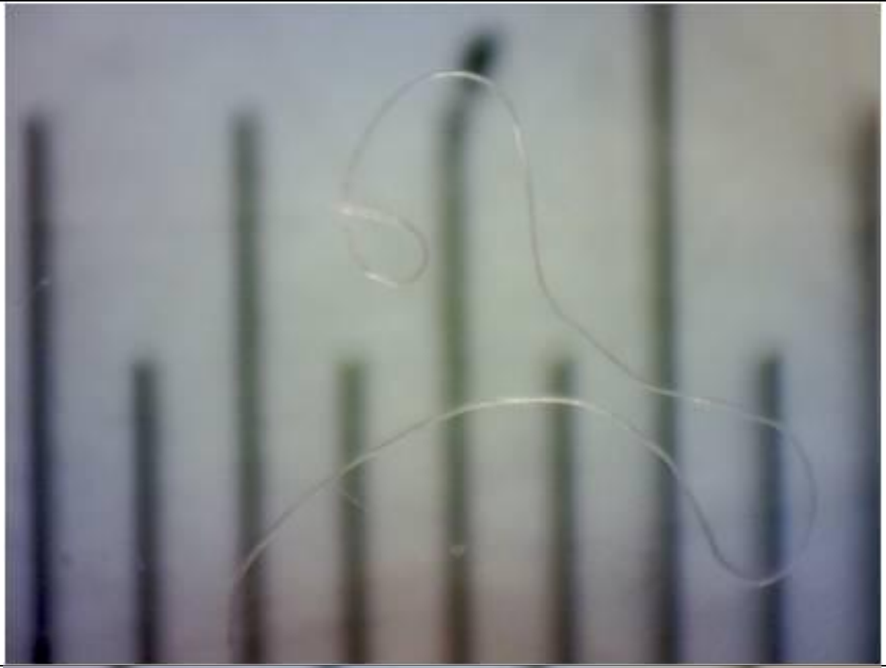
**Polyethylene**	**PE**	**87.3**		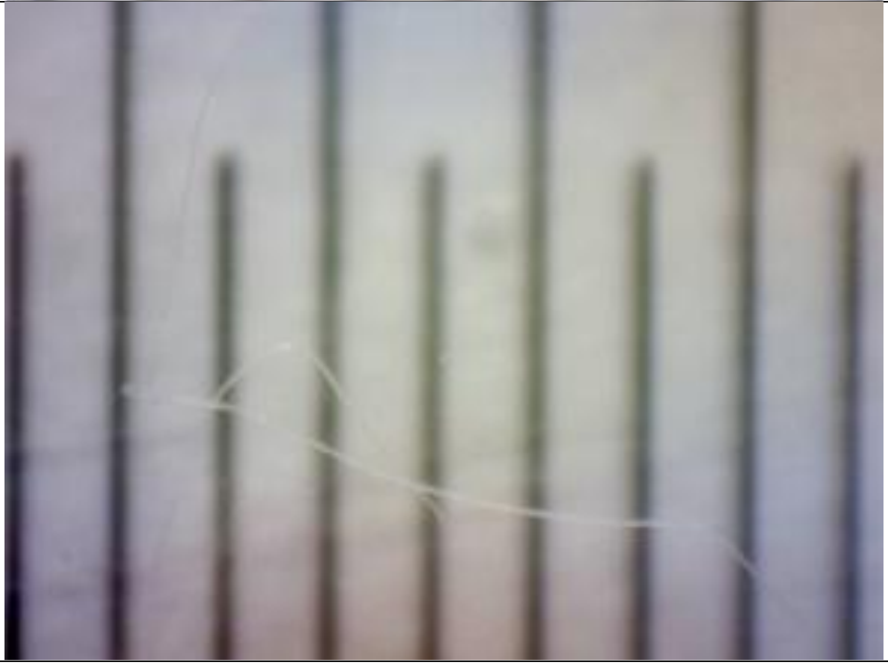

**Polymethylphenylsiloxane**	**PMPS**	**85.1**		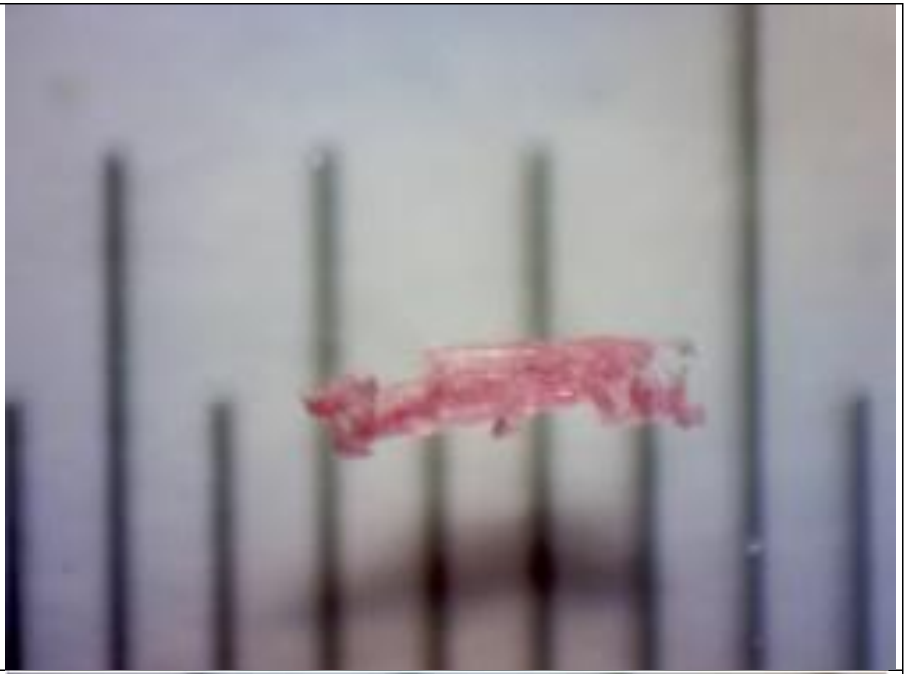
**Strong background fluorescence**	**fluorescence**			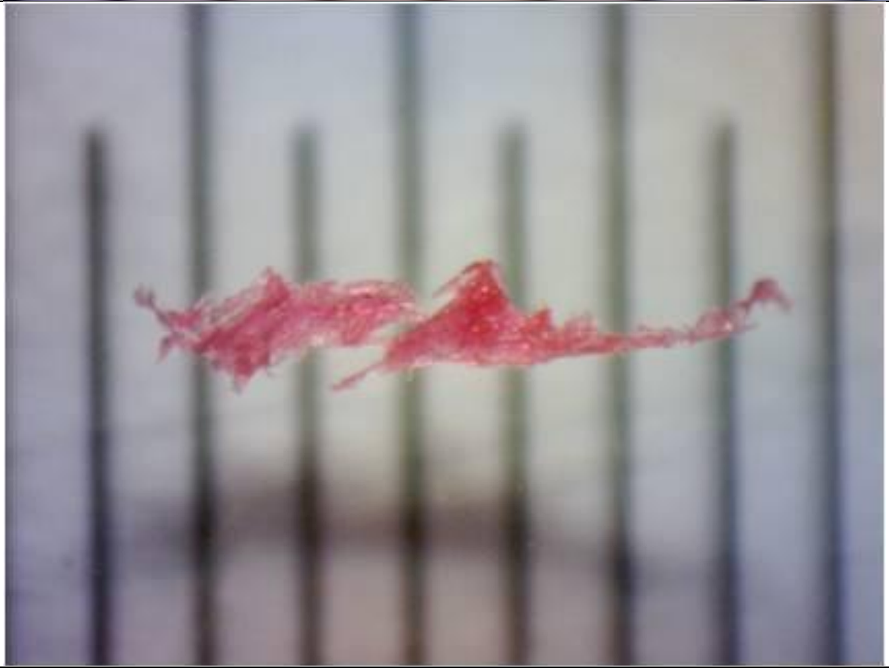

	**SD**	**95.5**	**Horasol Green G-K**	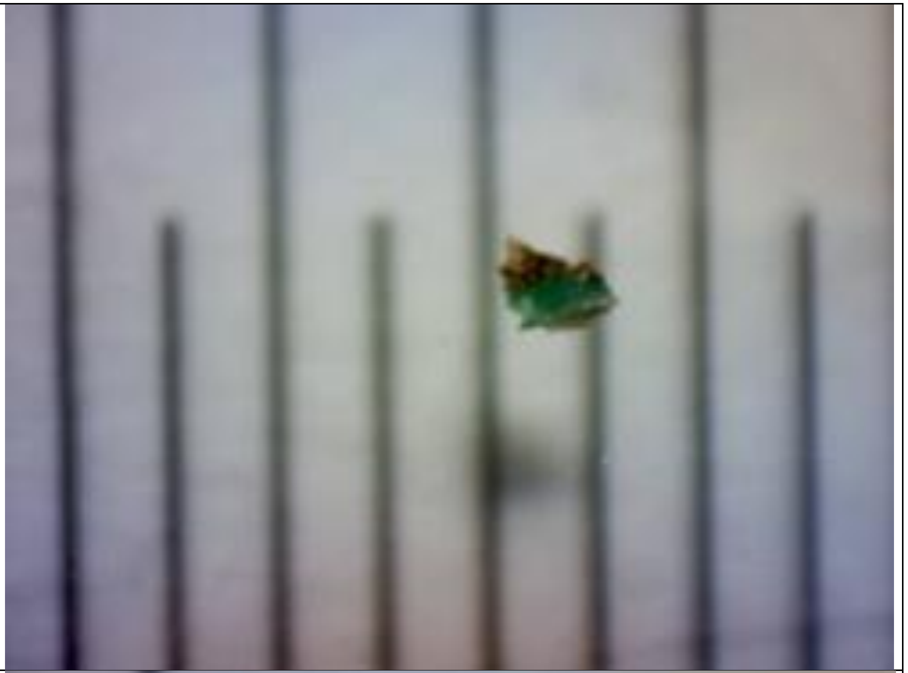
**Polyvinylidene chloride**	**PVDC**	**90.7**		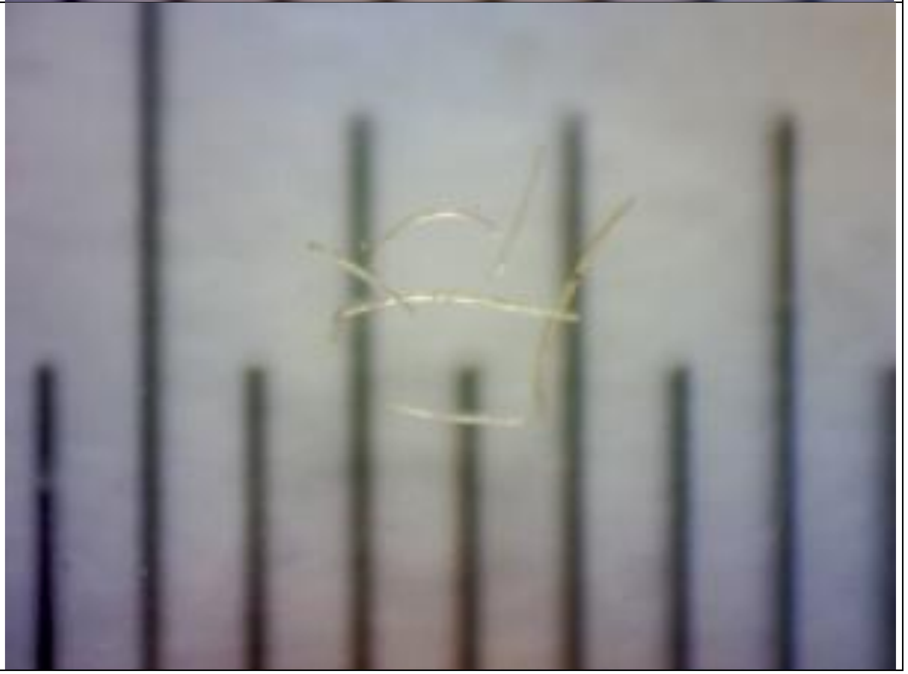

**Polypropylene**	**PP**	**85.1**	**Amido Black 10B**	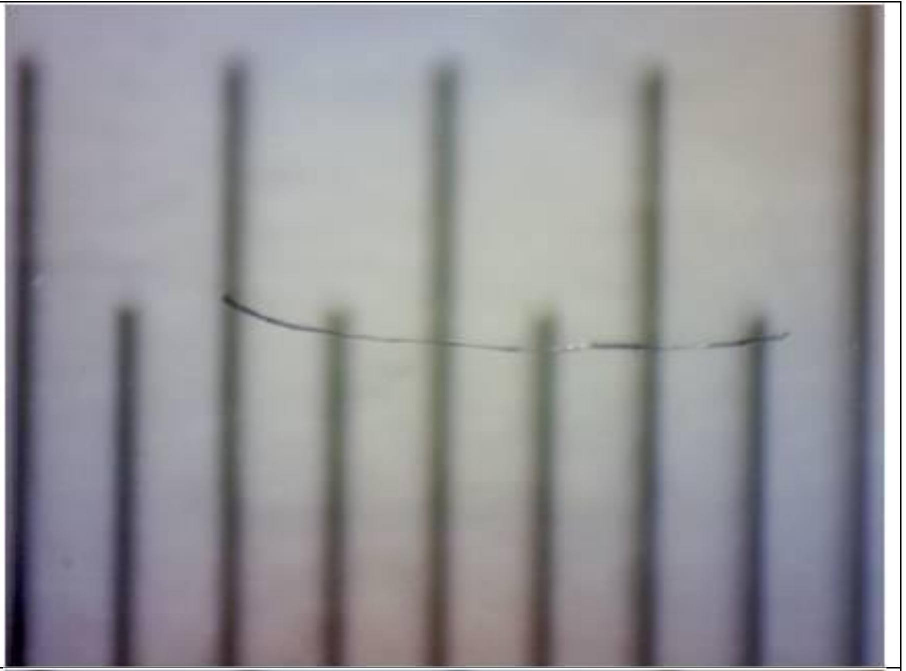
**Polystyrene**	**PS**	**87**		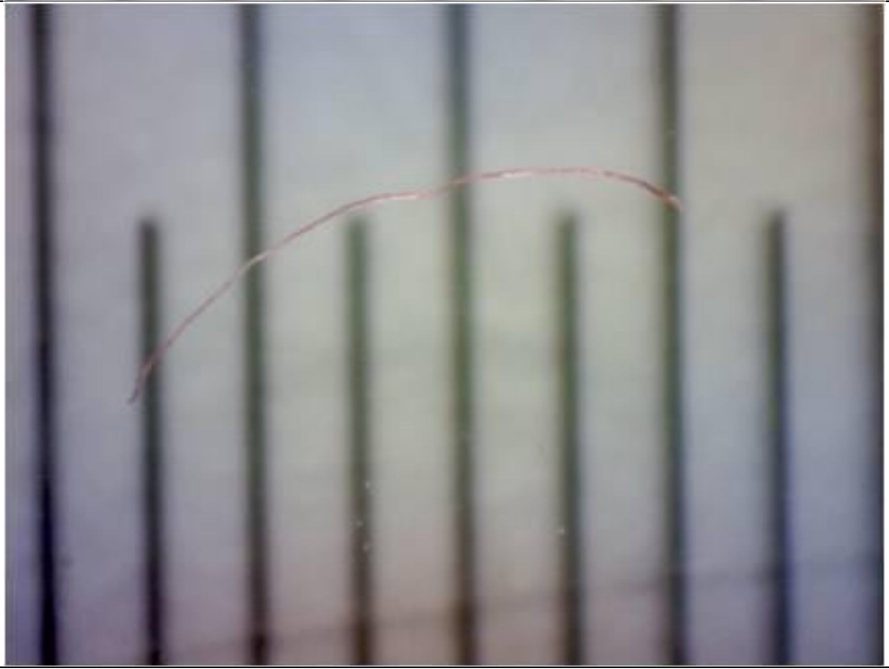

**Polyethylene (STAMYLAN 1000)**	**PE**	**94.4**		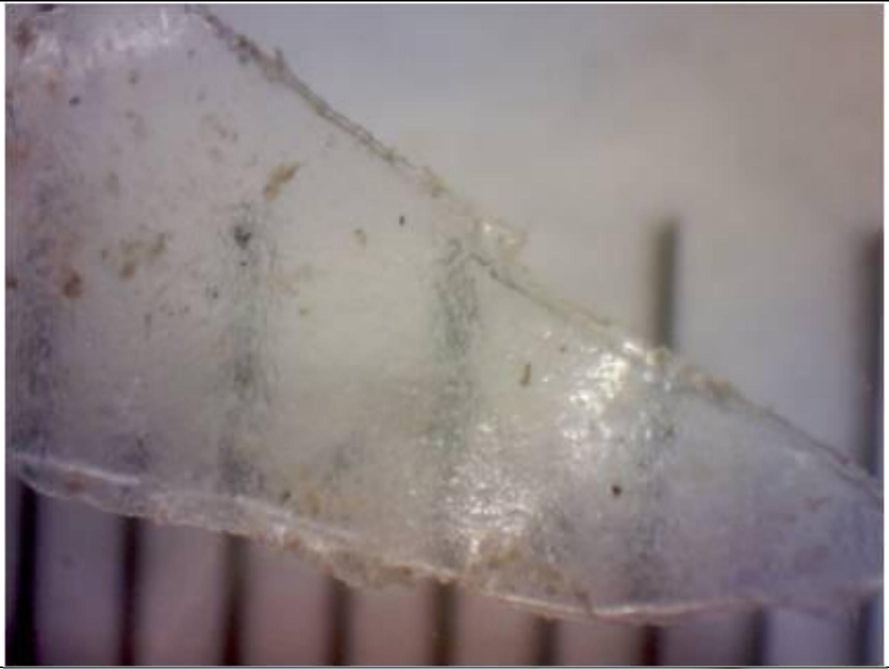
**Polyethylene**	**PE**	**86.8**		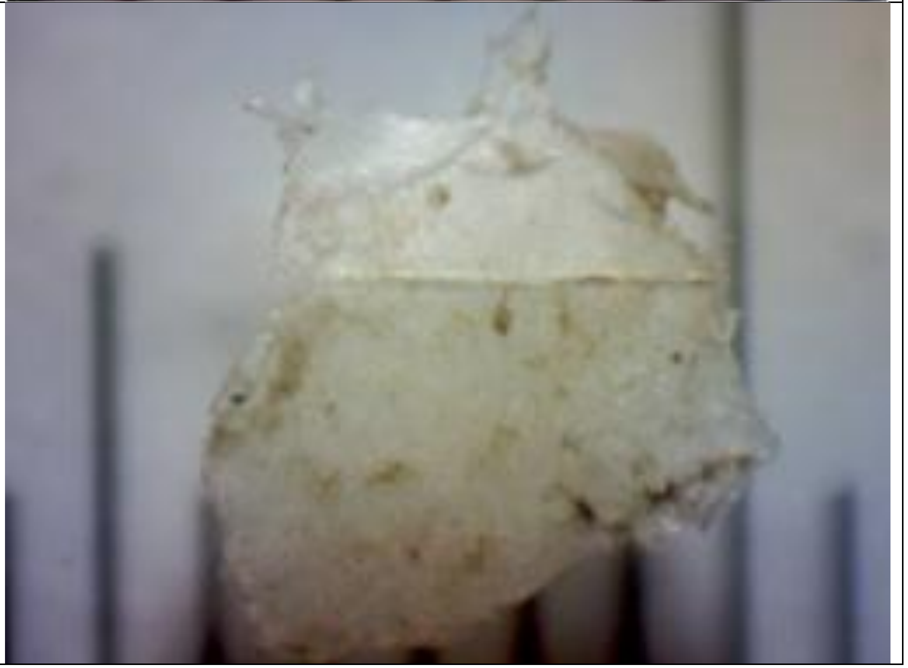

**Polypropylene**	**PP**	**85.8**	**Cobalt phthalocyanine**	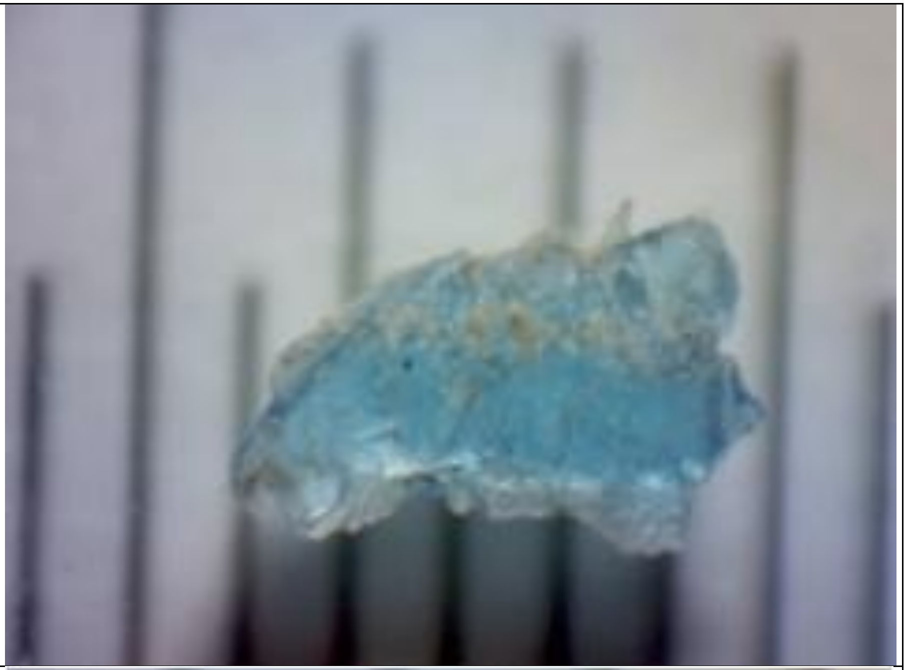
**Polypropylene**	**PP**	**98.2**		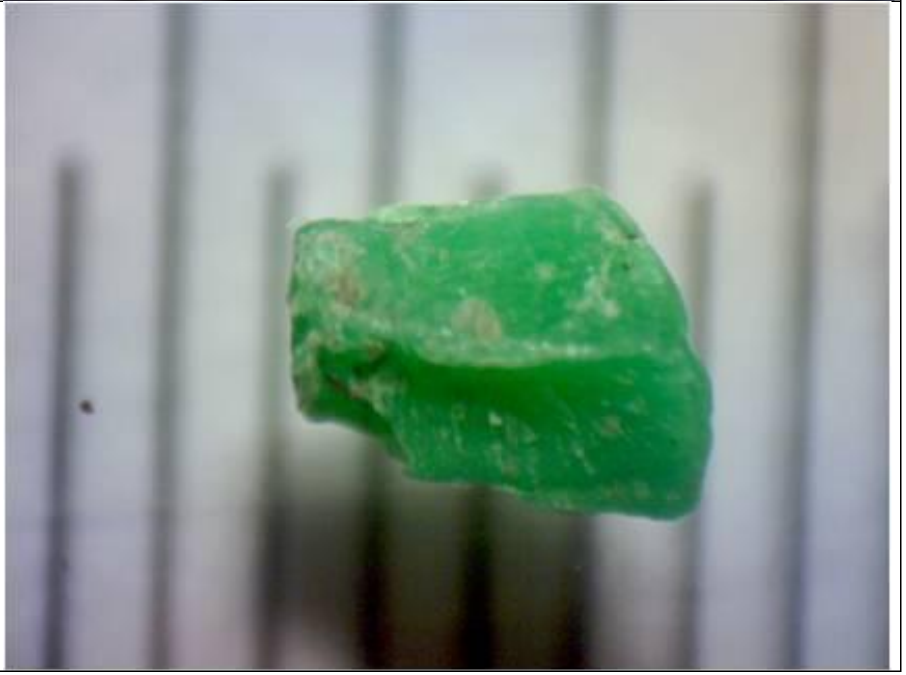

**Polypropylene**	**PP**	**67.8**		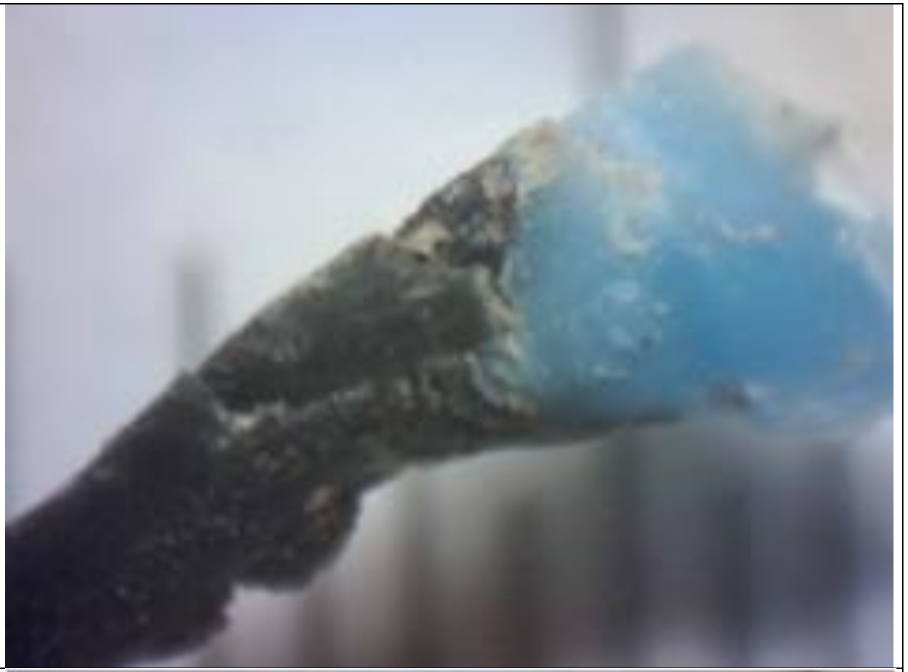
**Polyethylene**	**LDPE**	**97.3**		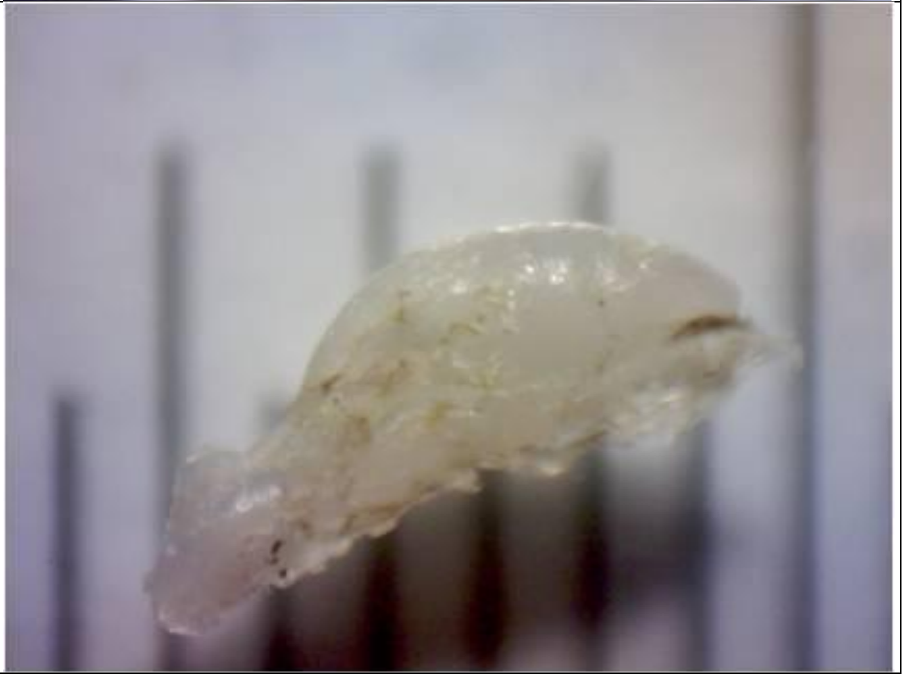

**Polyethylene**	**PE**	**92**		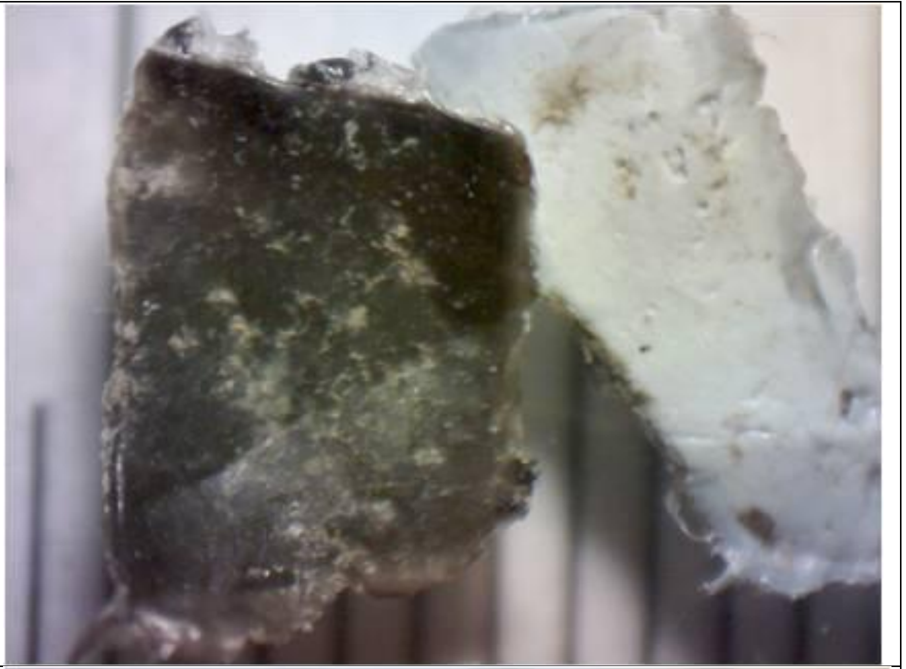
**Plastic wax**	**Plastic wax**	**87.9**		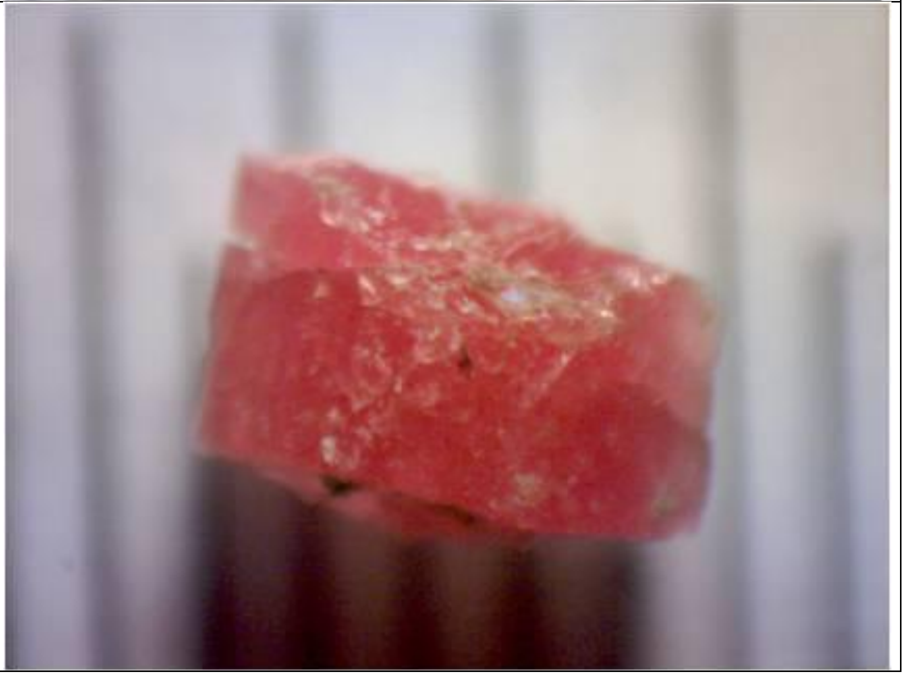

	SD	66.1	Motoperm Blue	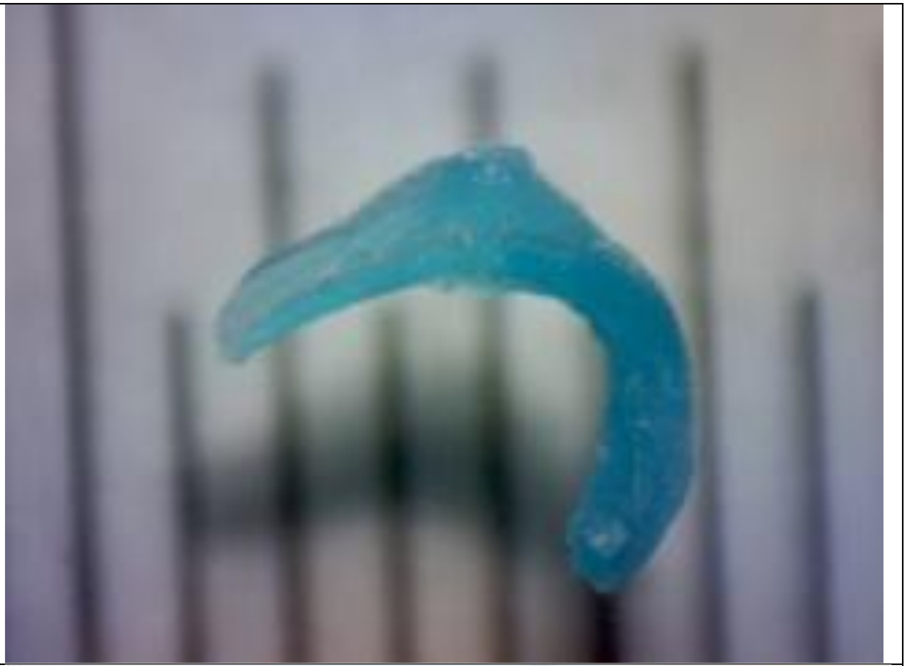
Polyethylene	PE	95.7		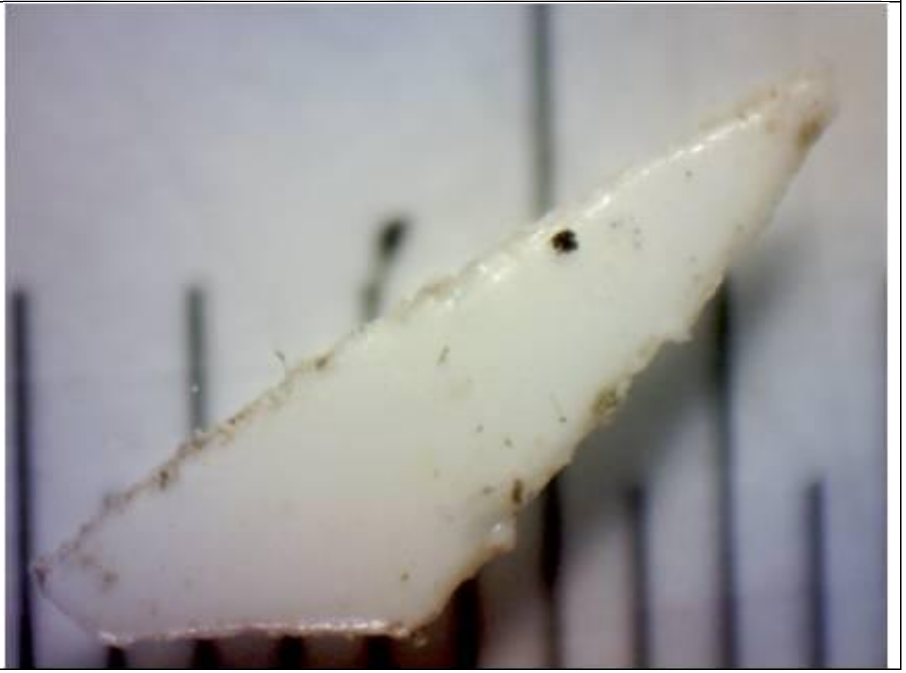

**Polypropylene**	**PP**	**94.9**		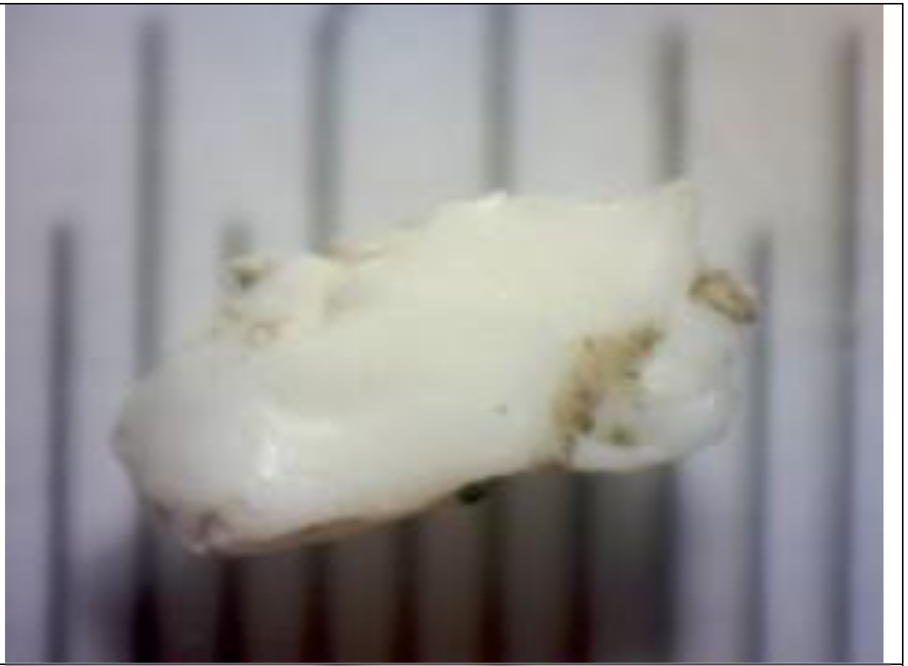
**Polypropylene**	**PP**	**90.2**		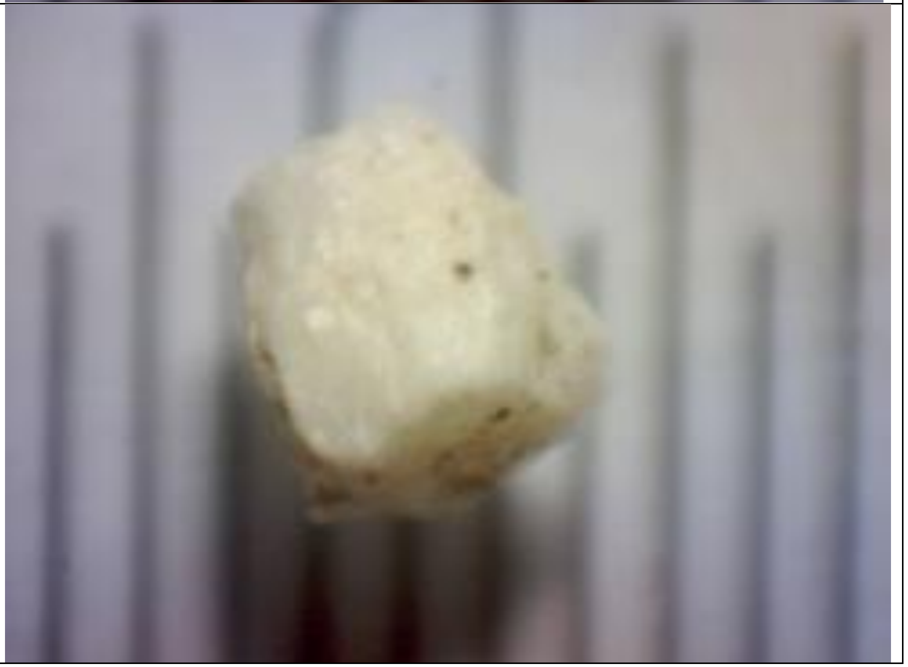

**Polyethylene**	**PE**	**97.2**		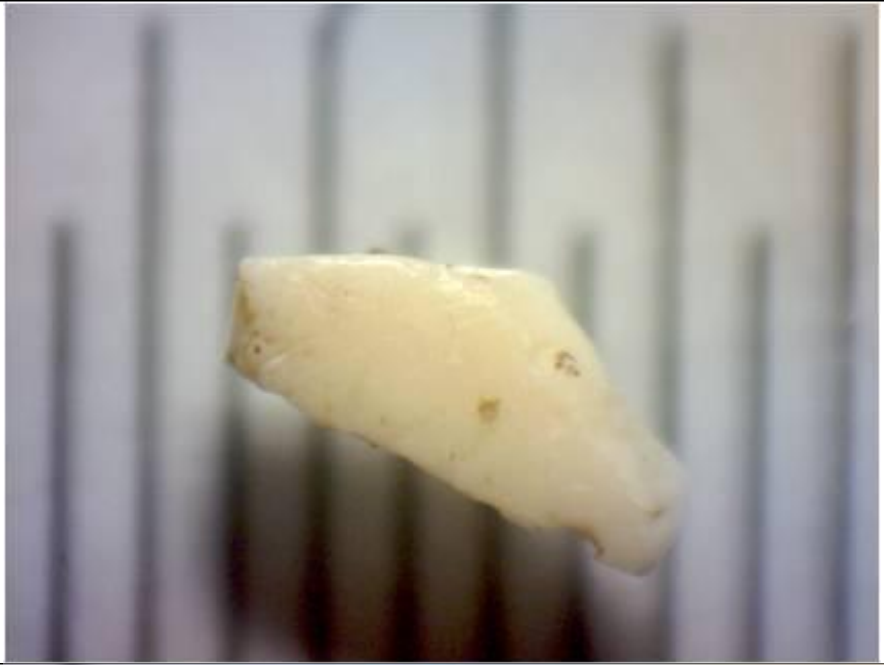
**Polyethylene**	**PE**	**84.9**		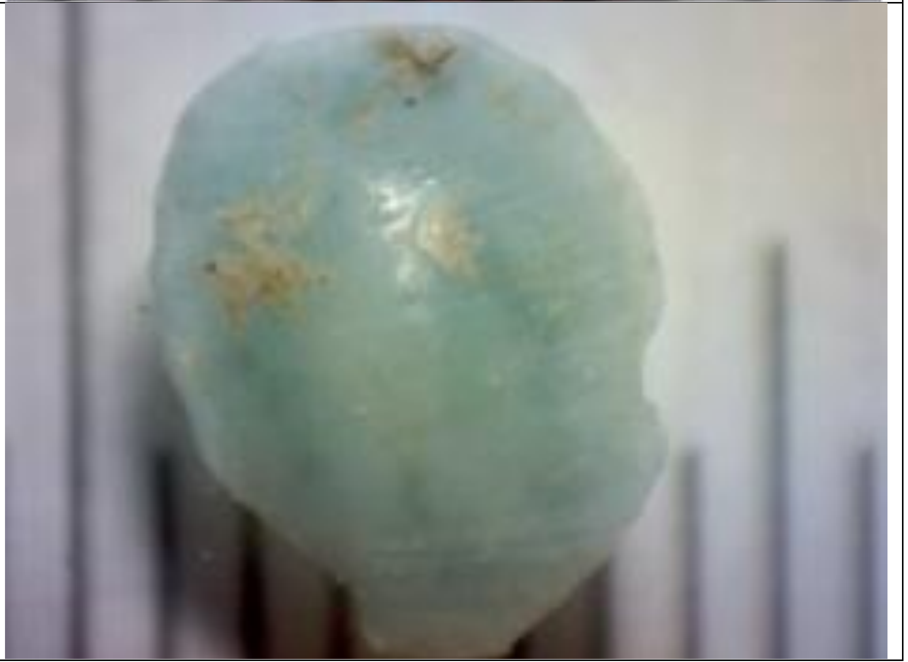

**Polyethylene**	**PE**	**92**		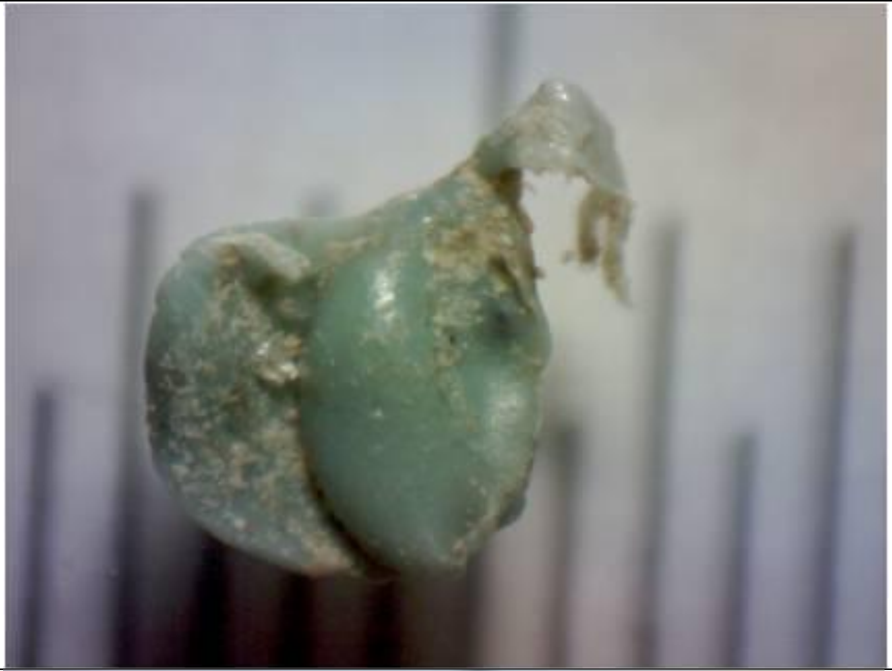
**Polypropylene**	**PP**	**86.5**		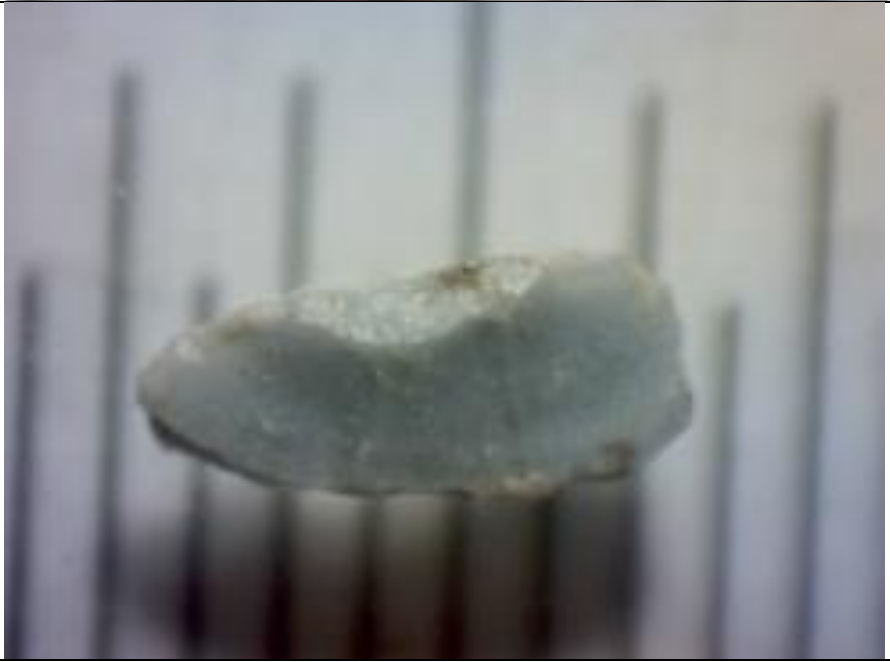

**Polyethylene**	**PE**	**94.3**	**Astra Blue Base**	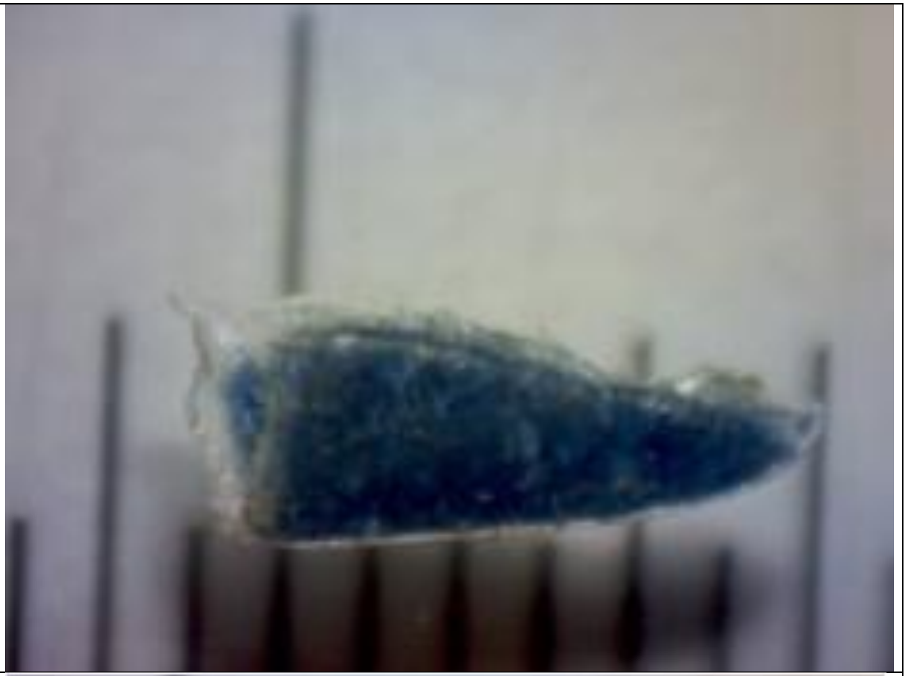
**Strong background fluorescence**	**fluorescence**			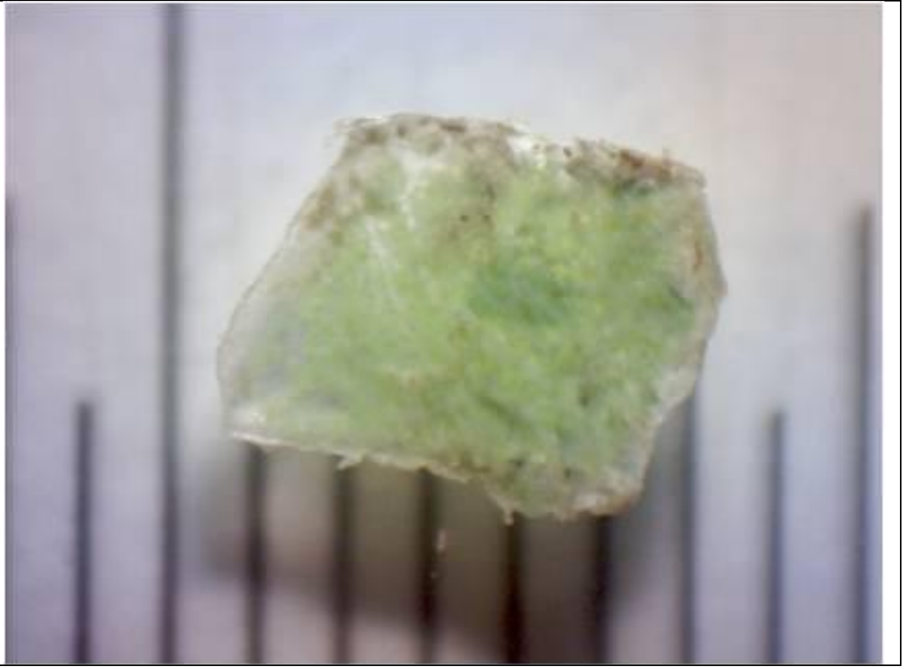

**Polyethylene**	**PE**	**88.3**		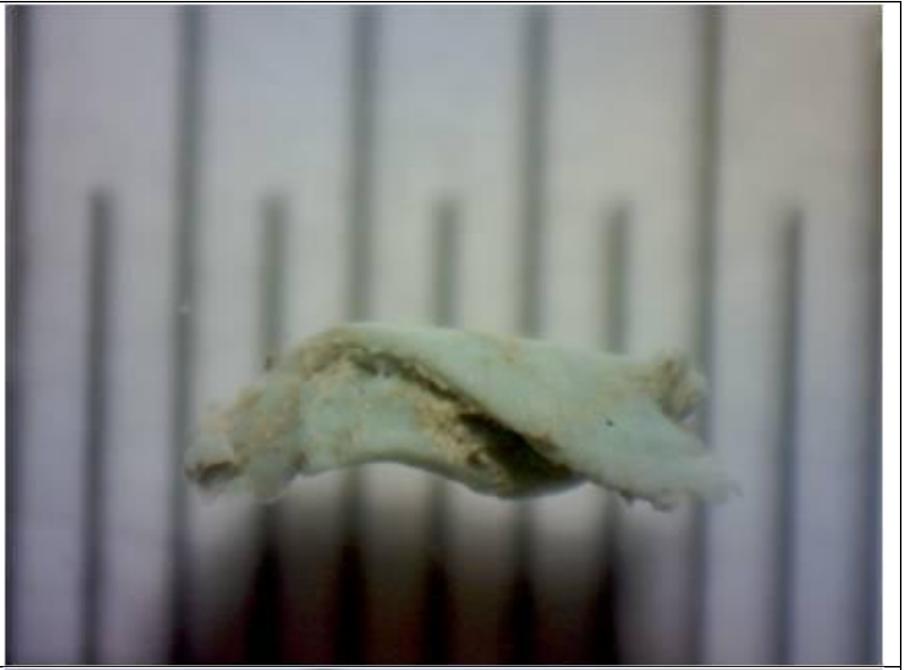
**Polyethylene**	**LDPE**	**98.3**		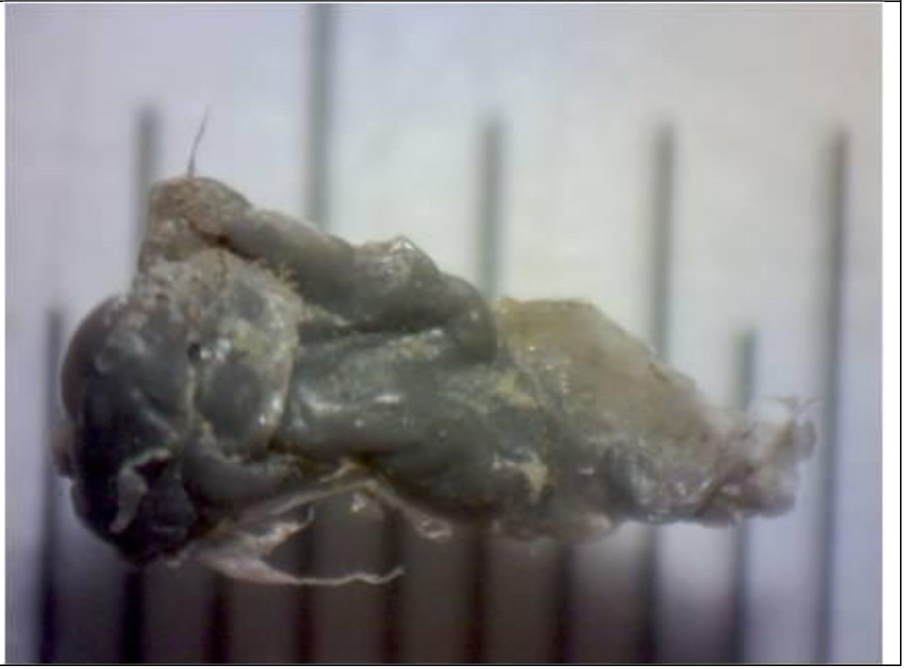

**Polyethylene + Polyvinyl butyral**	**PE + PVB**	**89.7 / 67.8**		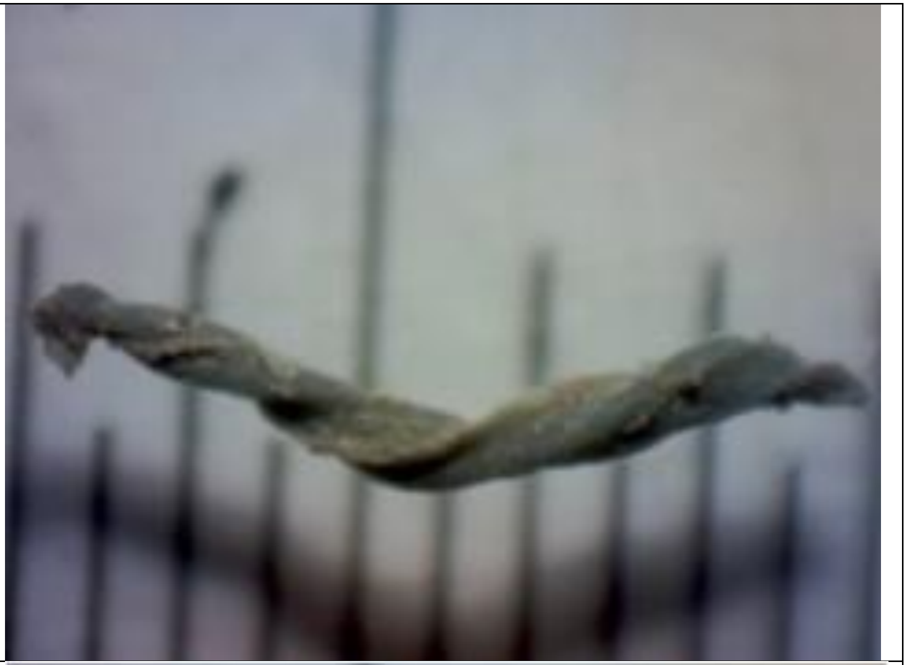
**Strong background fluorescence**	**fluorescen ce**			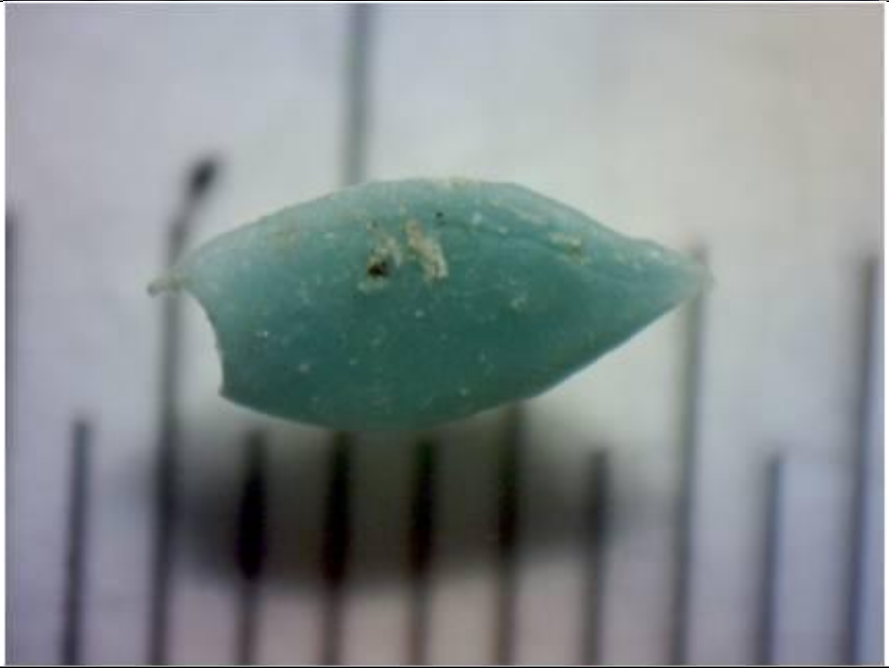

**Polyvinyl Butiral**	**PVB**	**70.5**	**Ingazin Blue**	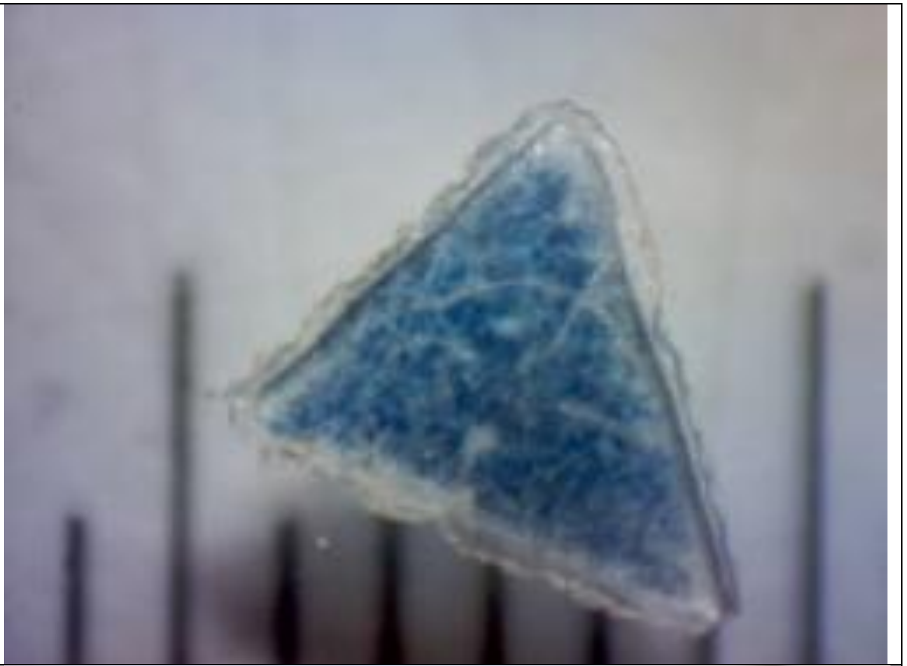
**Polypropylene**	**PP**	**81.2**		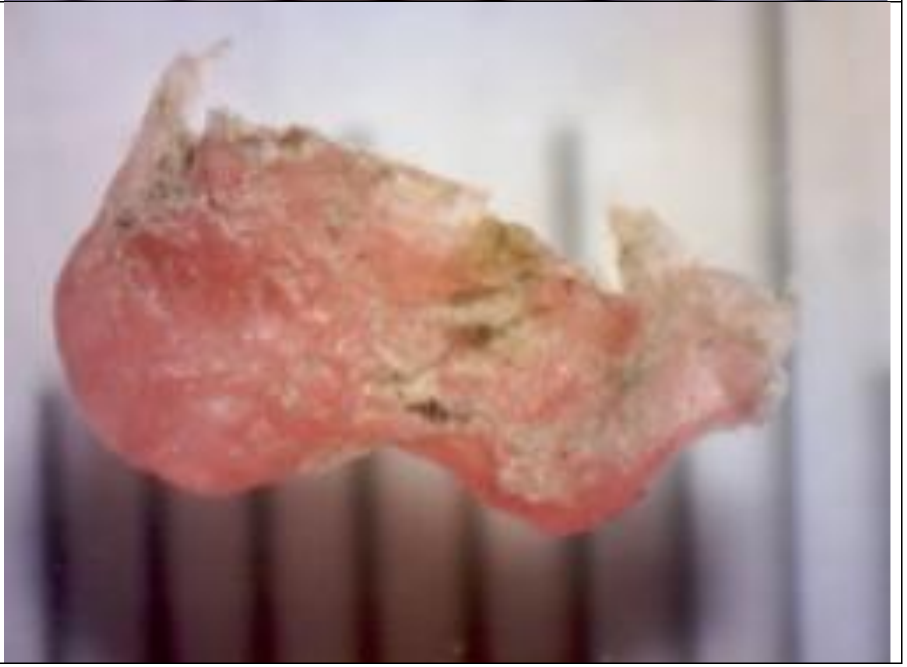

**Polyethylene**	**PE**	**74.8**		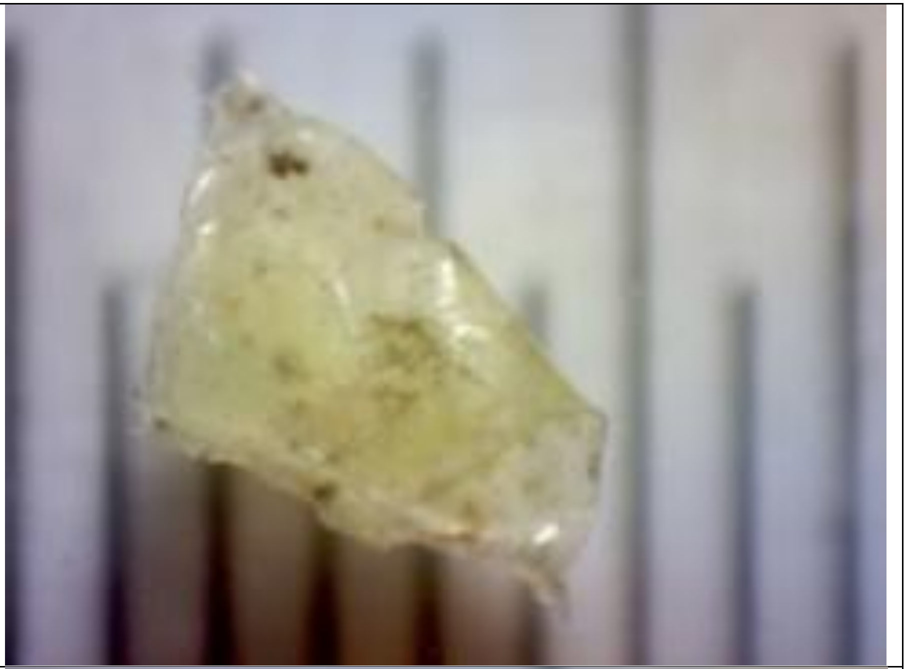
**Cellulose**	**CE**	**73.2**		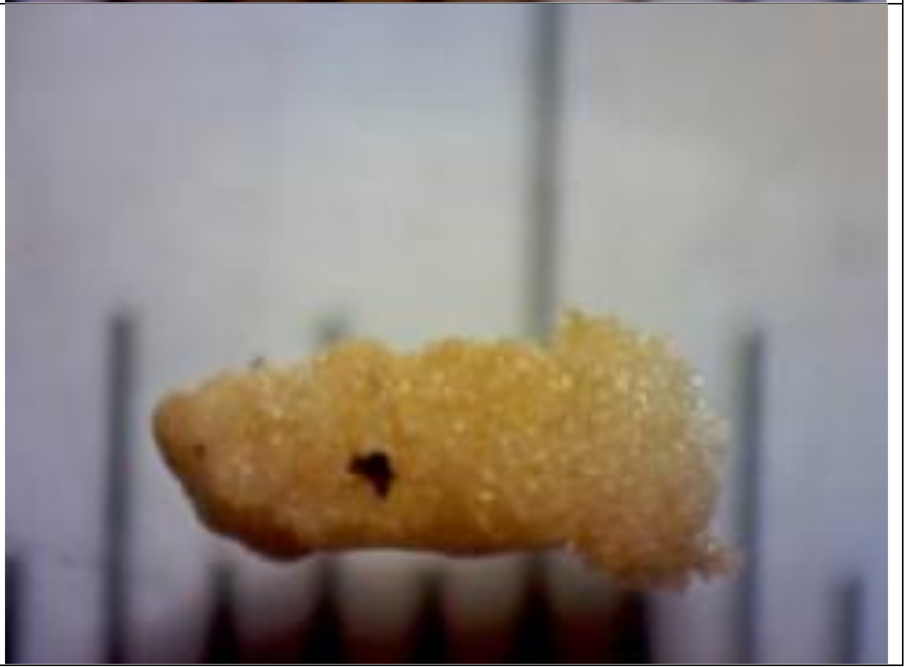

**Nylon 6**	**Nylon**	**91.2**		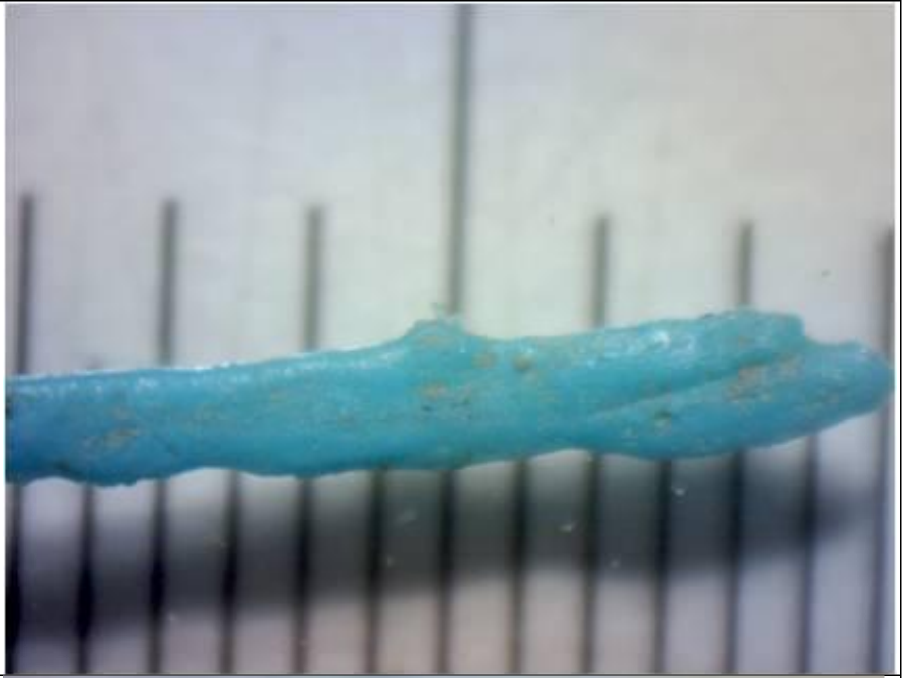
**Polyethylene + Polyvinyl butyral**	**PE + PVB**	**88.1 / 77.8**		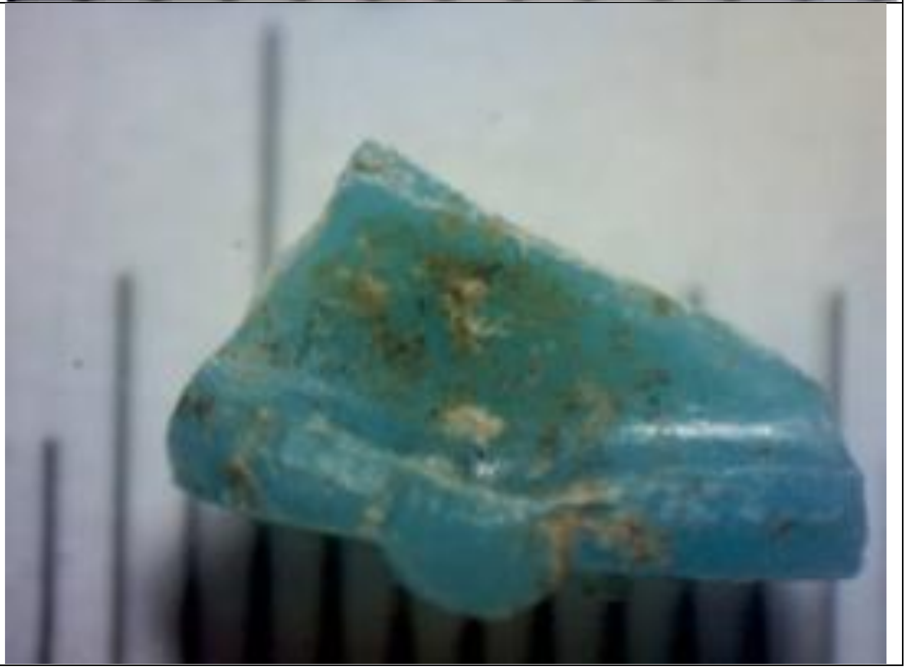

**Polyethylene**	**PE**	**95.9**		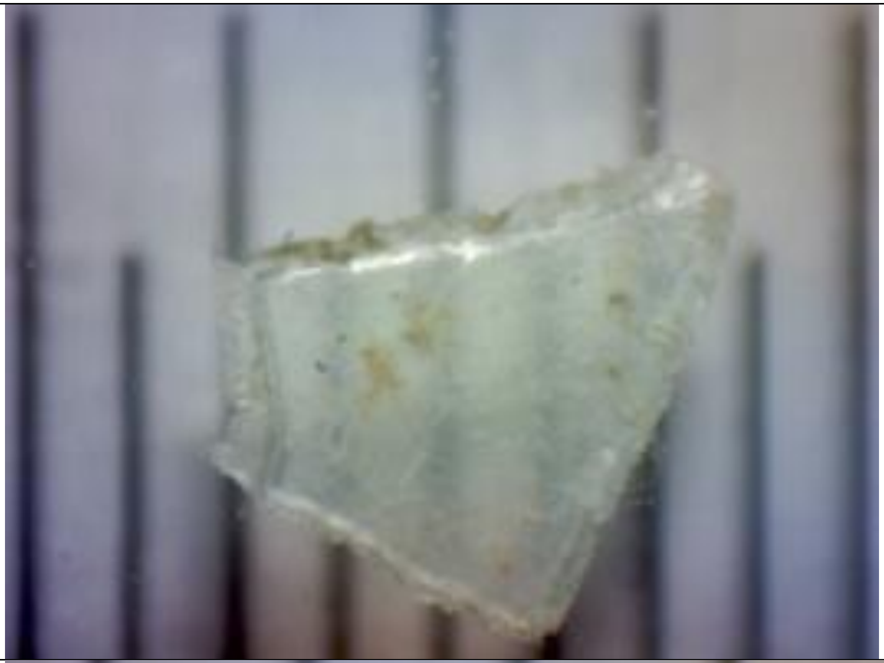
**Polyethylene**	**PE**	**62**	**Amido Black 10B**	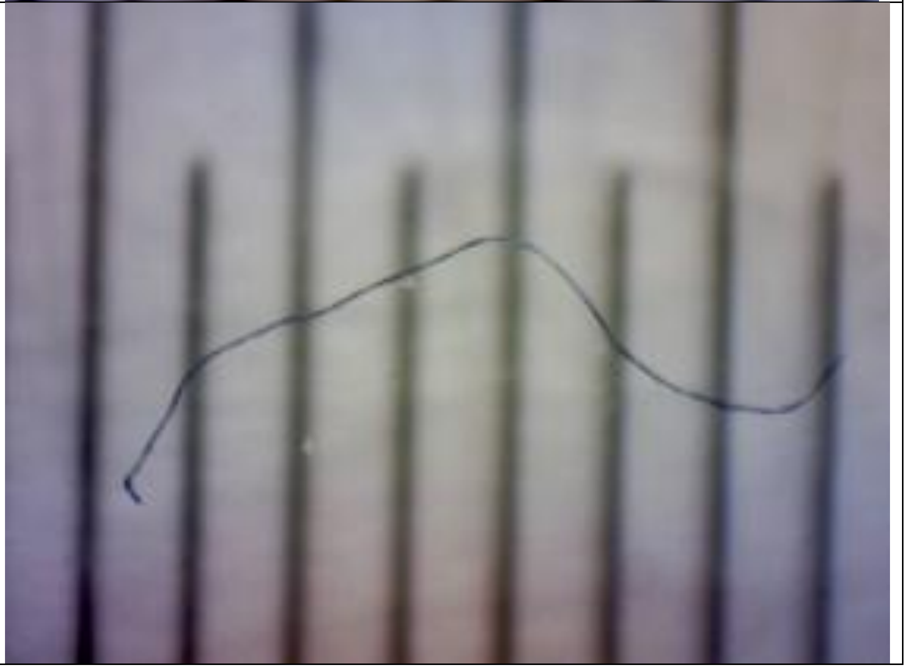

**Polypropylene**	**PP**	**69**		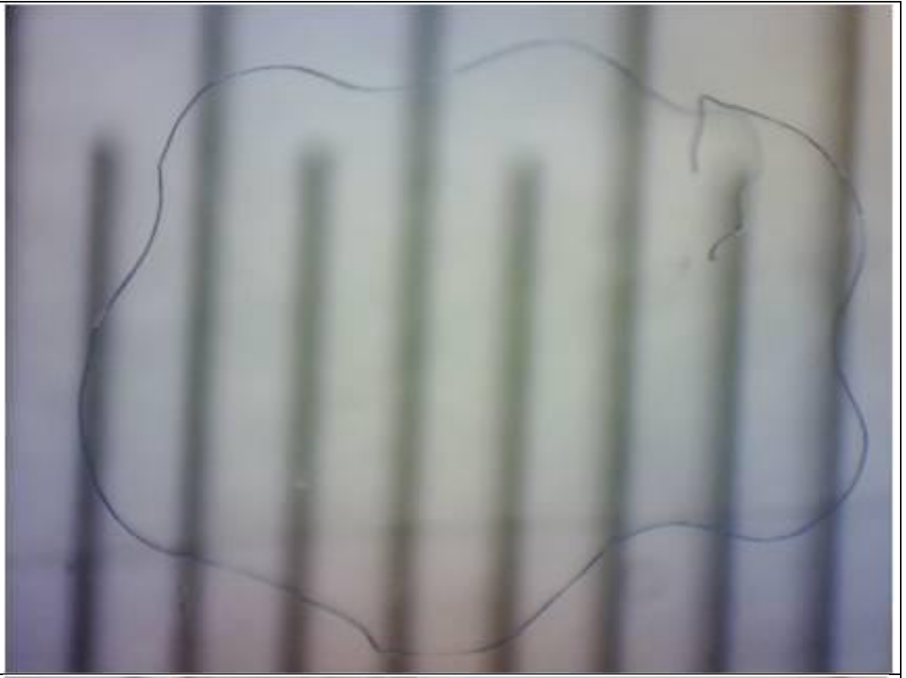
**Plastic wax**	**Plastic wax**	**92.3**		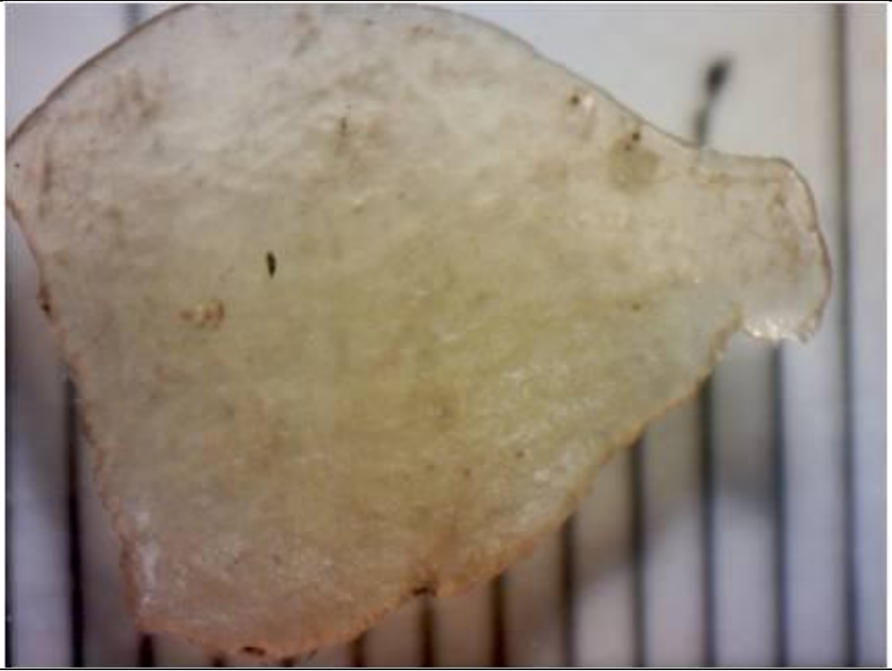

**Polyethylene**	**LDPE**	**91.6**		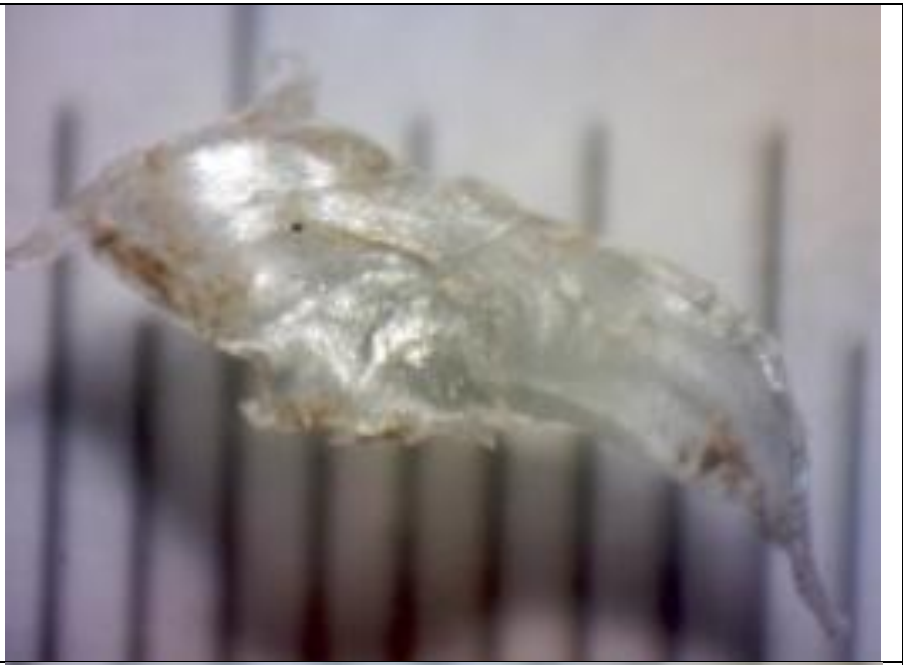
**Polyethylene**	**PE**	**83.5**		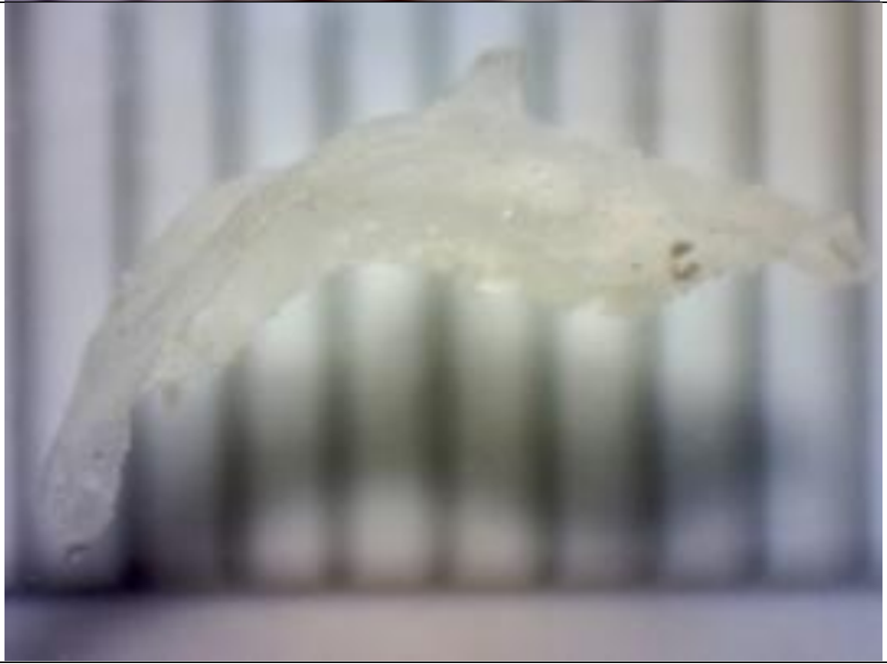

**Polypropylene (PolyPro F-975 D-modified PP)**	**PP**	**98.2**		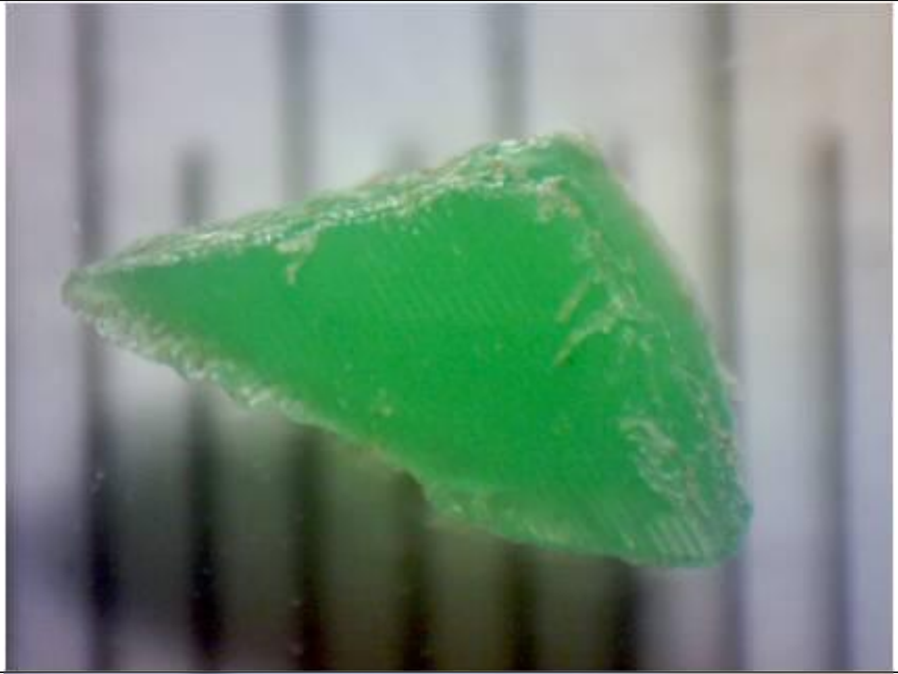
**Polyethylene**	**PE**	**91.3**		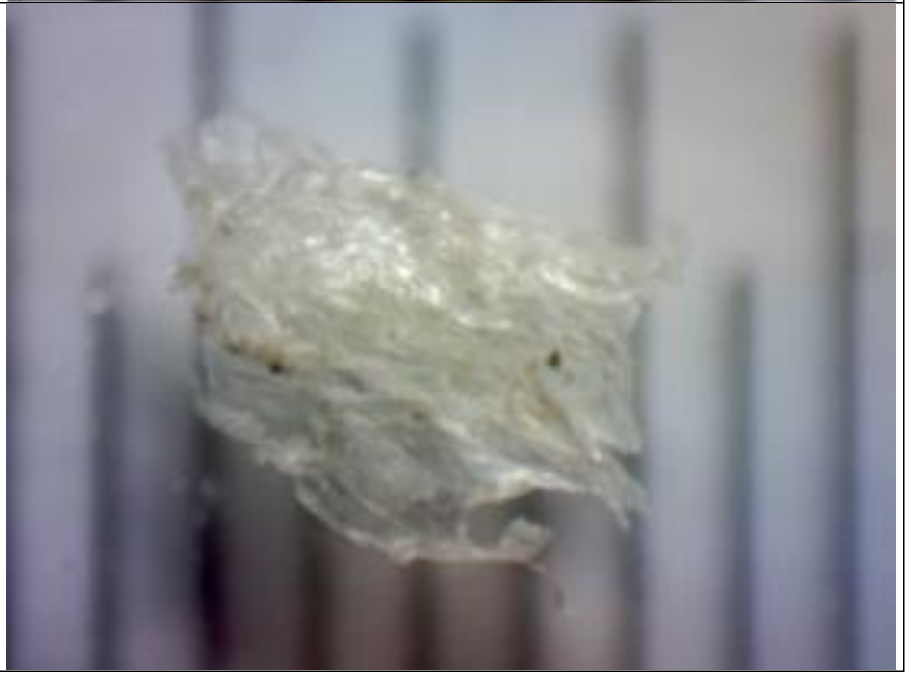

**Polyethylene**	**PE**	**94.7**		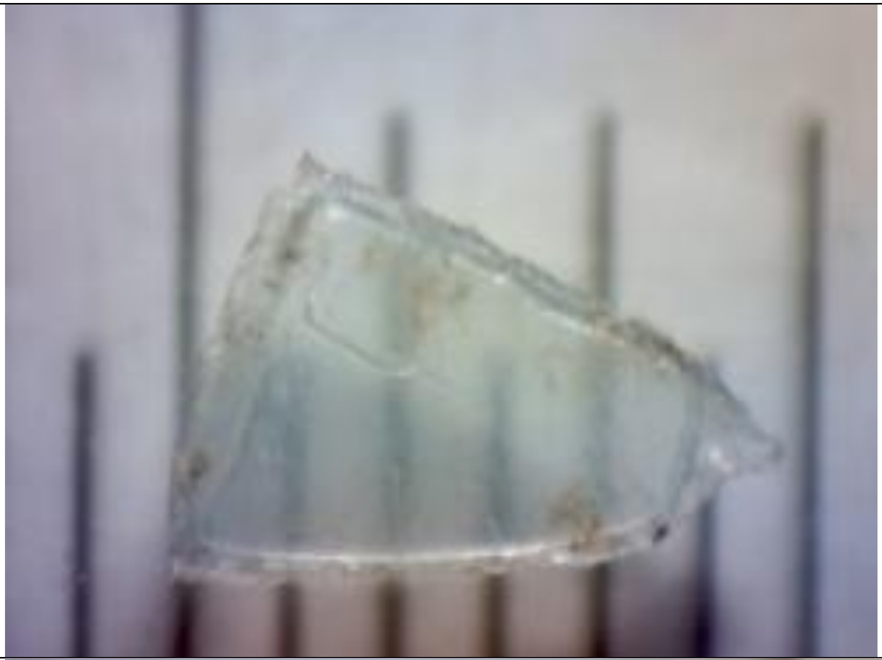
**Polyethylene**	**LDPE**	**87.3**		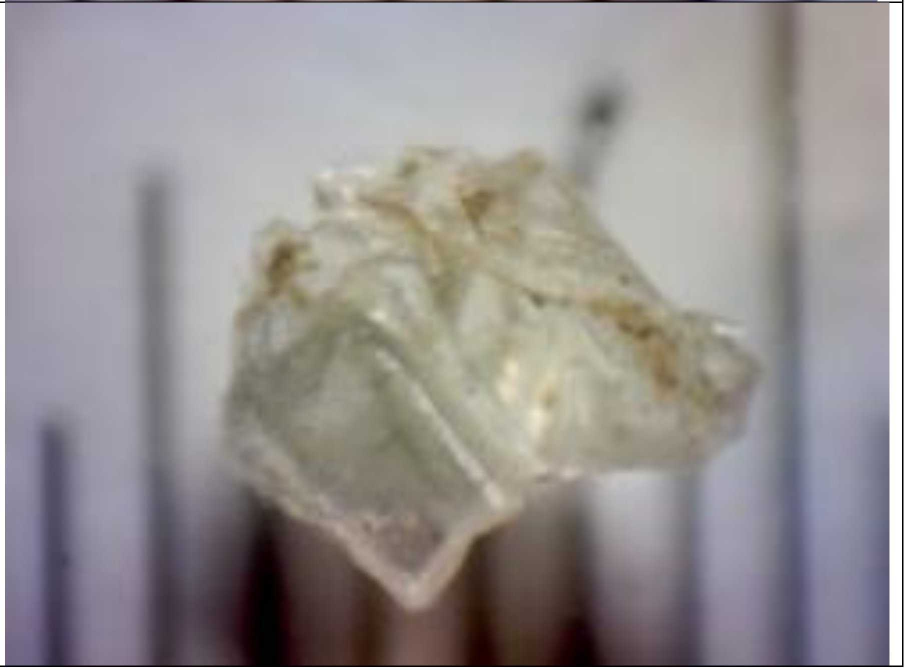

**Polyethylene**	**LDPE**	**98.3**		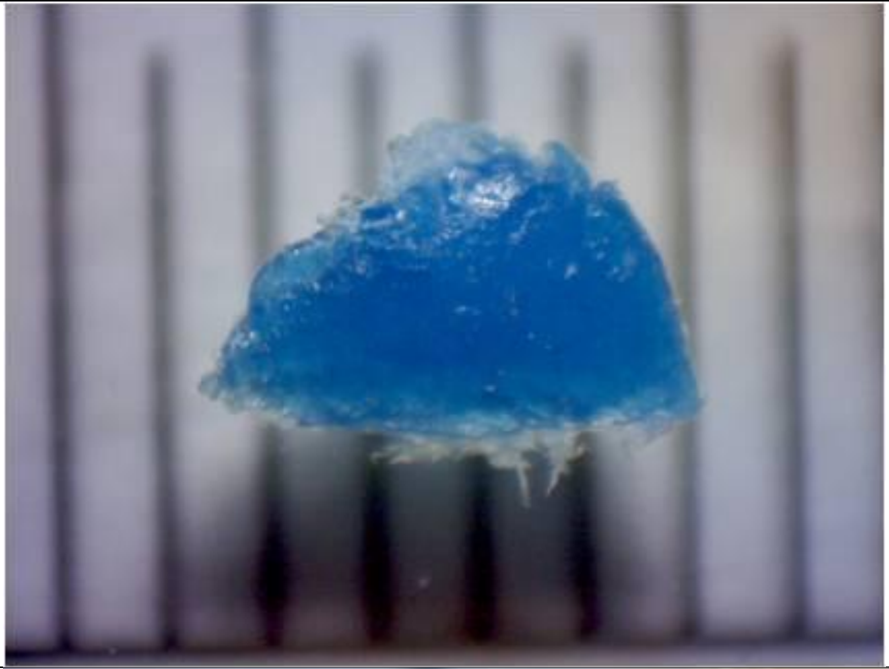
**Polyethylene**	**PE**	**85**		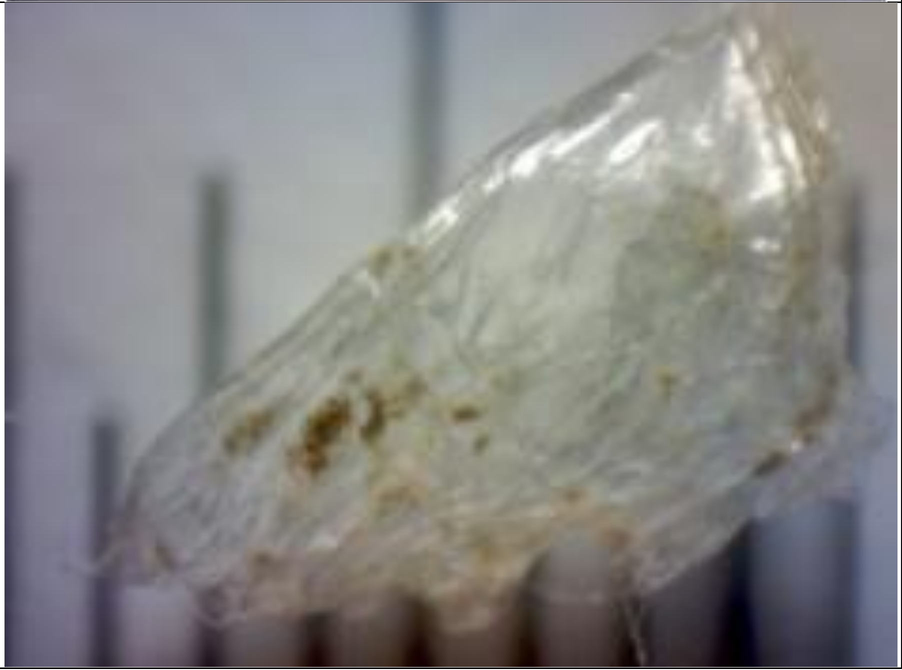

**Strong background fluorescence**	**fluorescen ce**			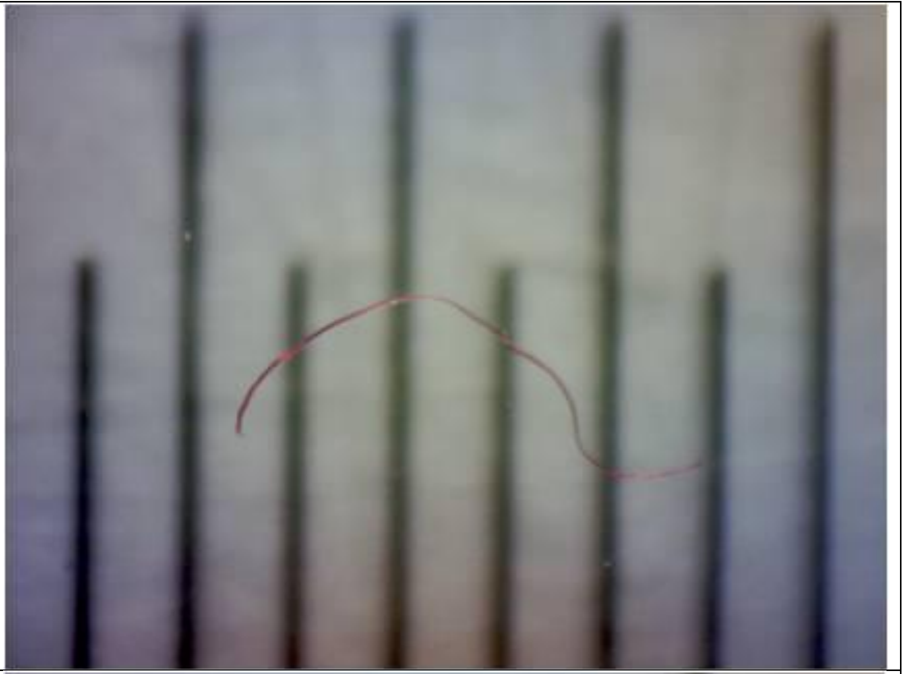
**Strong background fluorescence**	**fluorescen ce**			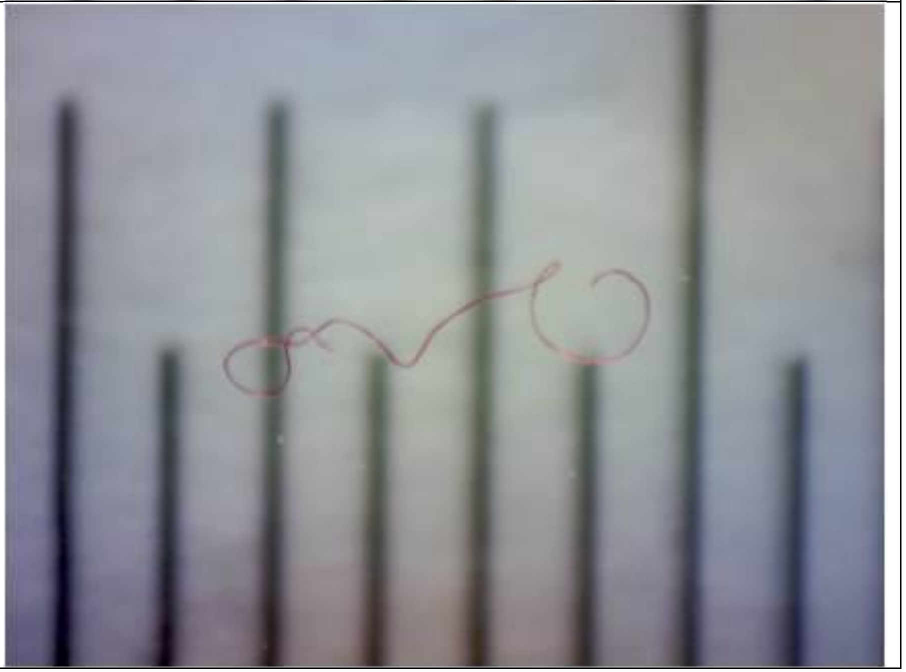

**Strong background fluorescence**	**fluorescence**			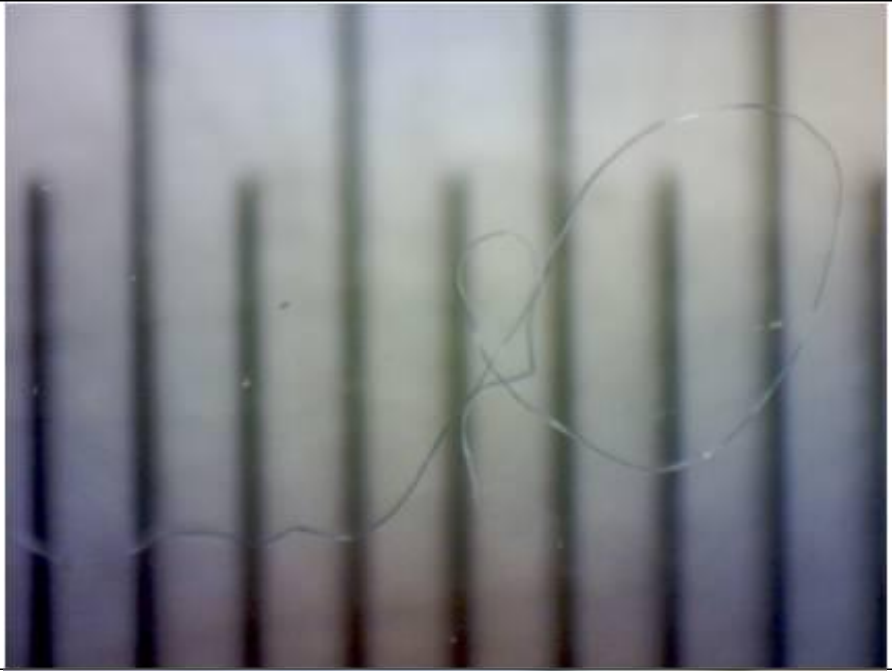
**Polyethylene**	**LDPE**	**93.2**		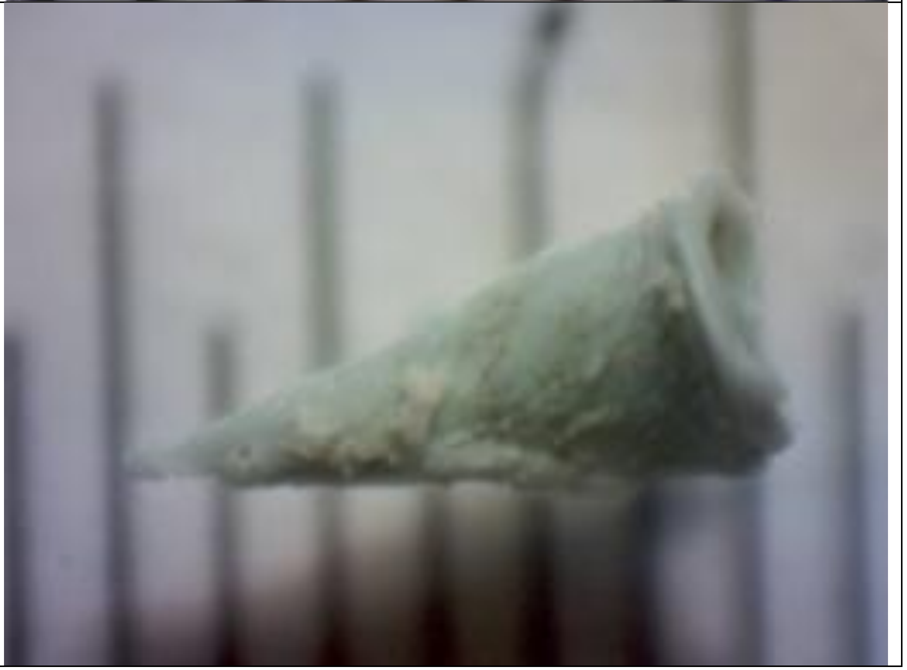

	**SD**	**85.7**	**Horasol Green G-K**	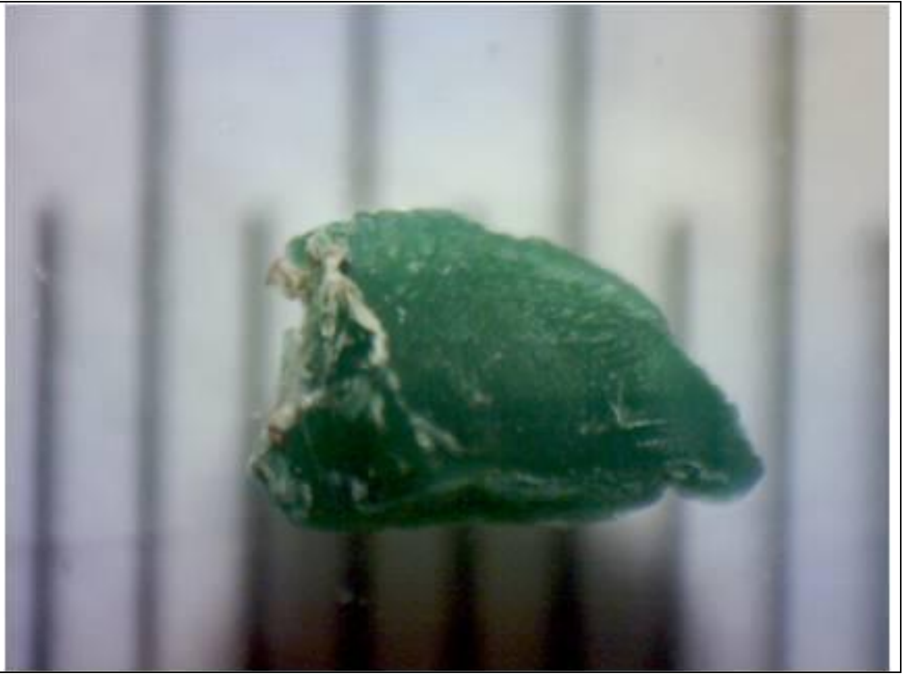
